# Xanthatin Targets CISD1 to Drive Ferroptosis and Mitophagy as a Dual Anticancer Strategy in Triple‐Negative Breast Cancer

**DOI:** 10.1002/advs.202520051

**Published:** 2026-02-06

**Authors:** Qinwen Liu, Haojie Chen, Xiang Li, Jingxin Liu, Yiwen Li, Zhenyi Shi, Shenshen Guo, Qingfeng Du, Aiping Lu, Daogang Guan

**Affiliations:** ^1^ Neurosurgery Center Department of Cerebrovascular Surgery Engineering Technology Research Center of Education Ministry of China on Diagnosis and Treatment of Cerebrovascular Disease Zhujiang Hospital Southern Medical University Guangzhou P. R. China; ^2^ Department of Biochemistry and Molecular Biology School of Basic Medical Sciences Southern Medical University Guangzhou P. R. China; ^3^ Institute of Systems Medicine and Health Sciences School of Chinese Medicine Hong Kong Baptist University Hong Kong P. R. China; ^4^ Guangdong Basic Research Center of Excellence for Integrated Traditional and Western Medicine For Qingzhi Diseases Guangzhou P. R. China; ^5^ School of Traditional Chinese Medicine Southern Medical University Guangzhou Guangdong P. R. China; ^6^ Southern Medical University Hospital of Integrated Traditional Chinese and Western Medicine Southern Medical University Guangzhou Guangdong P. R. China; ^7^ Guangdong Provincial Key Laboratory of Chinese Medicine Pharmaceutics Guangzhou Guangdong P. R. China; ^8^ Guangdong Provincial Key Laboratory of Single‐cell and Extracellular Vesicles Southern Medical University Guangzhou P. R. China

**Keywords:** CISD1, ferroptosis, mitochondrial autophagy, triple‐negative breast cancer, Xanthatin

## Abstract

Triple‐negative breast cancer (TNBC) is an aggressive subtype with poor prognosis. Here, we identify xanthatin, a sesquiterpene lactone from Xanthium species, as a potent inhibitor of TNBC cell growth with minimal toxicity to normal cells. Transcriptomic analyses revealed that xanthatin activates ferroptosis, evidenced by elevated ROS, lipid peroxidation, and Fe^2^^+^ accumulation, together with GSH depletion and downregulation of SLC7A11 and GPX4. Target identification by drug affinity responsive target stability and mass spectrometry uncovered CDGSH iron sulfur domain 1 (CISD1) as the direct binding partner of xanthatin. Cellular thermal shift assay, surface plasmon resonance, and dynamics simulations consistently demonstrated that tryptophan‐75 is the critical residue mediating this interaction. Functionally, xanthatin promotes CISD1 ubiquitination and proteasomal degradation, thereby disrupting mitochondrial iron homeostasis and inducing ferroptosis. CISD1 destabilization further impaired mitochondrial integrity and activated PINK1/Parkin‐dependent mitophagy, establishing a dual ferroptosis–mitophagy mechanism. Importantly, genetic knockdown of CISD1 markedly attenuated the anticancer activity of xanthatin, confirming its essential role. In an orthotopic TNBC mouse model, xanthatin significantly suppressed tumor growth without causing systemic toxicity. Collectively, our findings provide the first demonstration that xanthatin directly targets CISD1 at the Trp‐75 site to trigger ferroptosis and mitophagy, highlighting its promise as a therapeutic candidate for TNBC.

## Graphical abstract

Triple‐negative breast cancer has a poor prognosis. Xanthatin directly targets and degrades CISD1, triggering ferroptosis through iron dysregulation. The concurrently activated mitophagy synergistically amplifies ferroptotic cell death. Xanthatin also demonstrates potent in vivo anti‐tumor efficacy with minimal toxicity, highlighting its therapeutic potential against TNBC.

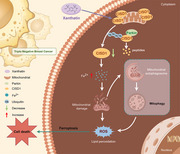

## 1 Introduction

Breast cancer is the most common malignancy and a leading cause of cancer‐related mortality among women worldwide [1]. Despite advances in screening and systemic therapies, it remains a heterogeneous disease with distinct biological subtypes that determine prognosis and treatment response [2]. Triple‐negative breast cancer (TNBC), defined by the absence of estrogen receptor (ER), progesterone receptor (PR), and human epidermal growth factor receptor 2 (HER2), accounts for 15–20% of all breast cancers and represents the most aggressive subtype [3]. TNBC is characterized by rapid progression, early recurrence, and frequent visceral and brain metastases, leading to poor clinical outcomes [4]. Unlike hormone receptor–positive or HER2‐amplified breast cancers, TNBC lacks validated molecular targets, leaving chemotherapy as the cornerstone of systemic treatment. Standard regimens such as anthracyclines, taxanes, and platinum agents can provide initial responses, but chemoresistance and systemic toxicity limit long‐term efficacy [4]. Although immune checkpoint inhibitors and antibody–drug conjugates have recently provided therapeutic opportunities, their benefits are confined to biomarker‐selected subsets such as PD‐L1–positive or HER2‐low patients [5]. Consequently, the overall prognosis of TNBC remains poor, underscoring the urgent need for novel small‐molecule therapeutics with high efficacy and low toxicity.

Natural products, as structurally diverse and biologically active small molecules derived from plants, microorganisms, and marine organisms, have historically provided an indispensable source of anti‐cancer drugs. Several clinically approved chemotherapeutics, such as paclitaxel from *Taxus brevifolia*, camptothecin derivatives from *Camptotheca acuminata*, and vinca alkaloids from *Catharanthus roseus*, originated from natural sources and continue to serve as cornerstones in oncology [6]. These examples highlight the therapeutic potential of natural compounds as scaffolds for drug discovery, particularly in challenging malignancies like TNBC, where effective treatment options are scarce. Xanthatin, a sesquiterpene lactone isolated from *Xanthium sibiricum* and related species, has recently attracted attention due to its reported pharmacological activities [7]. It has been reported to suppress tumor growth by activating CHOP‐mediated endoplasmic reticulum stress in glioma, [8] to induce apoptosis via reactive oxygen species (ROS) accumulation and caspase activation in retinoblastoma, [9] and to inhibit NF‐κB signaling in non‐small‐cell lung cancer [10]. Despite these promising observations, the precise molecular targets of xanthatin and its mode of action in TNBC have not been systematically elucidated. Whether xanthatin can overcome the therapeutic bottleneck of TNBC by engaging specific cell death pathways or modulating tumor‐associated signaling networks remains an open question.

CDGSH iron sulfur domain 1 (CISD1), also known as mitoNEET, is a [2Fe‐2S] cluster‐containing protein located on the mitochondrial outer membrane that plays an essential role in maintaining mitochondrial redox homeostasis and iron–sulfur cluster transfer [11]. Structurally, CISD1 has been identified as a potential drug target in metabolic and proliferative diseases due to its redox‐ and pH‐sensing capabilities [12]. Crucially, mounting clinical and experimental evidence implicates CISD1 as a significant prognostic biomarker across multiple cancer types. In breast cancer, elevated CISD1 expression has been correlated with shortened overall survival and enhanced immune infiltration, indicating its potential role in tumor progression and immune escape [13]. Similarly, high CISD1 expression has been associated with poor prognosis in lung adenocarcinoma [14] and several other malignancies [15]. Functionally, CISD1 overexpression enhances tumor cell proliferation by preserving mitochondrial integrity and mitigating oxidative stress, while its depletion triggers mitochondrial dysfunction and renders cells more susceptible to regulated cell death pathways [16]. Thus, CISD1 represents not only a marker of aggressive disease but also a mechanistic nexus linking mitochondrial stability with tumor vitality. In the context of TNBC, where therapy options are limited, targeting CISD1 could offer a novel strategy that exploits mitochondrial vulnerabilities in cancer cells.

In this study, we sought to clarify the anticancer mechanism of xanthatin in TNBC. Using integrative pharmacological and molecular approaches, we identified CISD1 as a direct functional target of xanthatin and further demonstrated that Trp‐75 is the critical residue mediating this interaction. We showed that xanthatin destabilizes CISD1 protein, thereby inducing ferroptosis and mitophagy, two interconnected mitochondrial stress pathways that drive tumor suppression. Functional assays confirmed that the antitumor effects of xanthatin, including inhibition of proliferation and induction of cell death, are dependent on CISD1 expression. Furthermore, in vivo experiments validated that xanthatin effectively restrains tumor growth in TNBC orthotopic models without apparent systemic toxicity. Collectively, our findings uncover CISD1 as a mitochondrial vulnerability in TNBC and establish xanthatin as a natural compound that exploits this vulnerability through Trp‐75–dependent binding and coordinated induction of ferroptosis and mitophagy.

## 2 Results

### 2.1 Xanthatin Inhibits Proliferation of TNBC Cells While Showing Low Toxicity to Breast Epithelial Cells

**FIGURE 1:**
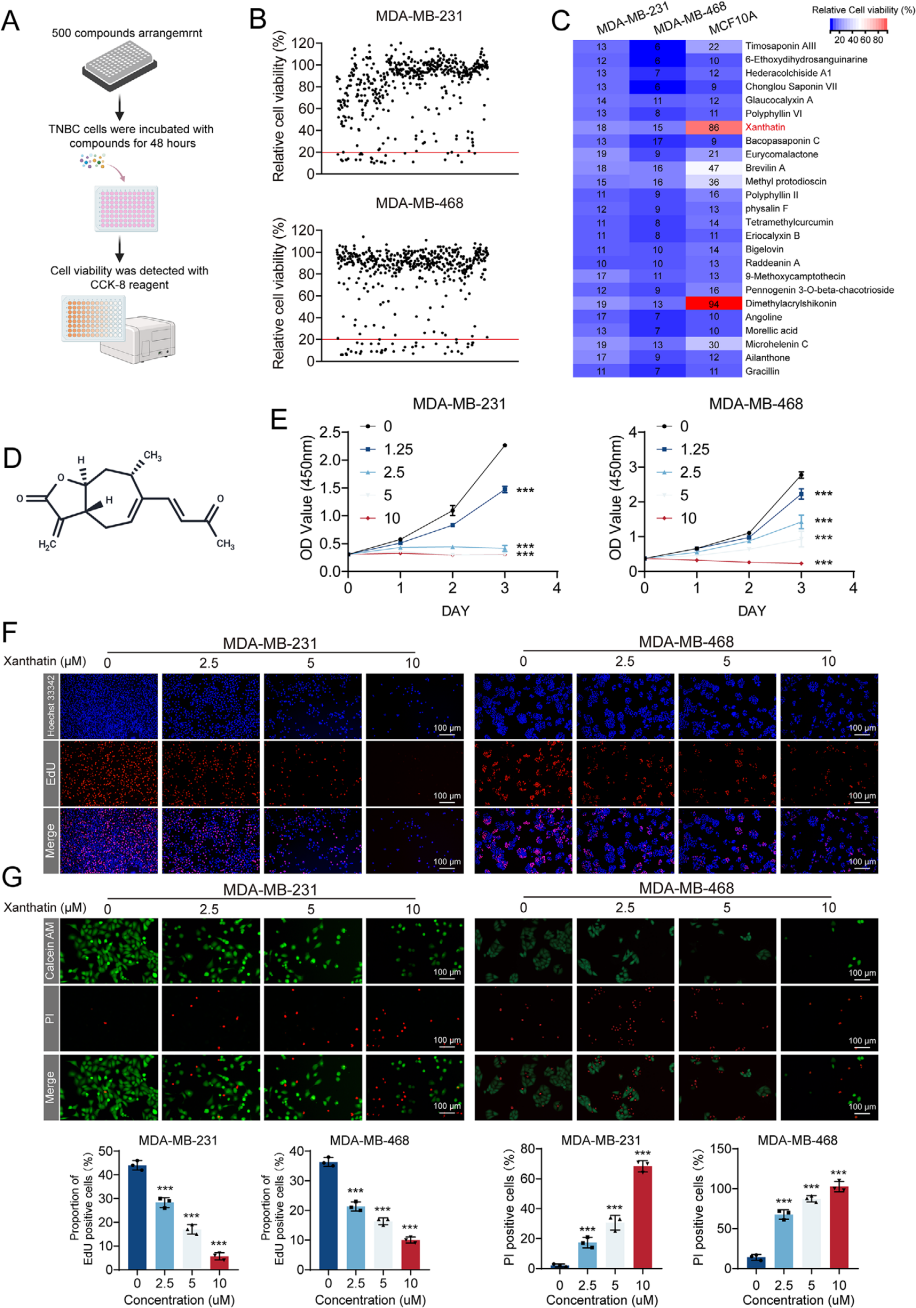
Xanthatin inhibits proliferation of TNBC cells while showing low toxicity to breast epithelial cells. (A) Schematic illustration of compound library screening workflow. TNBC cells were incubated with 500 natural compounds for 48 h, and cell viability was assessed using the CCK‐8 assay. (B) Scatter plots showing relative cell viability of MDA‐MB‐231 and MDA‐MB‐468 cells after compound treatment for 48 h. (C) Heatmap presenting the relative viability of TNBC cells and MCF10A after compound treatment for 48 h. (D) Chemical structure of xanthatin. (E) Growth curves of MDA‐MB‐231 and MDA‐MB‐468 cells treated with the indicated concentrations of xanthatin for indicated time. (F) Representative images of EdU incorporation assays and quantification of proliferating cells in MDA‐MB‐231 and MDA‐MB‐468 cells after treatment with xanthatin for 48 h. Scale bar = 100 µm. (G) Representative fluorescence images showing PI‐positive apoptotic cells (red) in MDA‐MB‐231 and MDA‐MB‐468 cells after treatment with xanthatin for 48 h. Scale bar = 100 µm. Bars, SDs; ^*^ 0.01< *p* < 0.05, ^**^ 0.001< *p* < 0.01, and ^***^ *p* < 0.001.

To identify natural compounds with selective anti‐TNBC activity, we performed a high‐throughput screen of a 500‐compound natural product library (Figure 1A). Cell viability was assessed by CCK‐8 assays after treatment of TNBC cells (MDA‐MB‐231 and MDA‐MB‐468) and normal breast epithelial cells (MCF10A) with each compound at a concentration of 10 µm for 48 h (Figure 1B; Table S1). Among the screened molecules, 25 compounds reduced TNBC cell viability by more than 80% (cell viability <20%; Figure 1C). However, most of these candidates also showed considerable cytotoxicity toward MCF10A cells. Notably, xanthatin exhibited a strong inhibitory effect on TNBC cells while exerting negligible toxicity on MCF10A cells, thus emerging as the most promising candidate for subsequent studies (Figure 1C,D). To further characterize its anti‐cancer potential, MDA‐MB‐231 and MDA‐MB‐468 cells were exposed to increasing concentrations of xanthatin for up to 3 days, followed by CCK‐8 analysis. The results demonstrated a significant suppression of cell viability in a dose‐ and time‐dependent manner (Figure 1E). Notably, the half‐maximal inhibitory concentration (IC_50_) values further quantified its potency and selectivity: Xanthatin exhibited IC_50_ values of 2.45 and 5.63 µm against MDA‐MB‐231 and MDA‐MB‐468 cells, respectively, whereas the IC_50_ for MCF10A cells was as high as 53.9 µm, demonstrating a favorable selectivity (Figure S1A). Consistently, EdU incorporation assay revealed that DNA synthesis was strongly impaired in xanthatin‐treated cells, reflected by a progressive reduction in EdU‐positive nuclei (Figure 1F). Long‐term proliferative capacity was also compromised, as evidenced by the marked decrease in colony numbers in colony formation assays (Figure S1B). In addition to proliferation inhibition, we evaluated whether xanthatin exerts direct cytotoxicity on TNBC cells. Live/dead staining indicated that with increasing concentrations of xanthatin, calcein‐AM–positive viable cells (green fluorescence) decreased, whereas PI‐positive dead cells (red fluorescence) accumulated correspondingly (Figure 1G). Collectively, these findings clearly establish xanthatin as a potent anti‐TNBC agent with selective cytotoxicity toward cancer cells and minimal effects on normal breast epithelial cells.

### 2.2 Xanthatin Induces Ferroptosis in TNBC Cells

**FIGURE 2:**
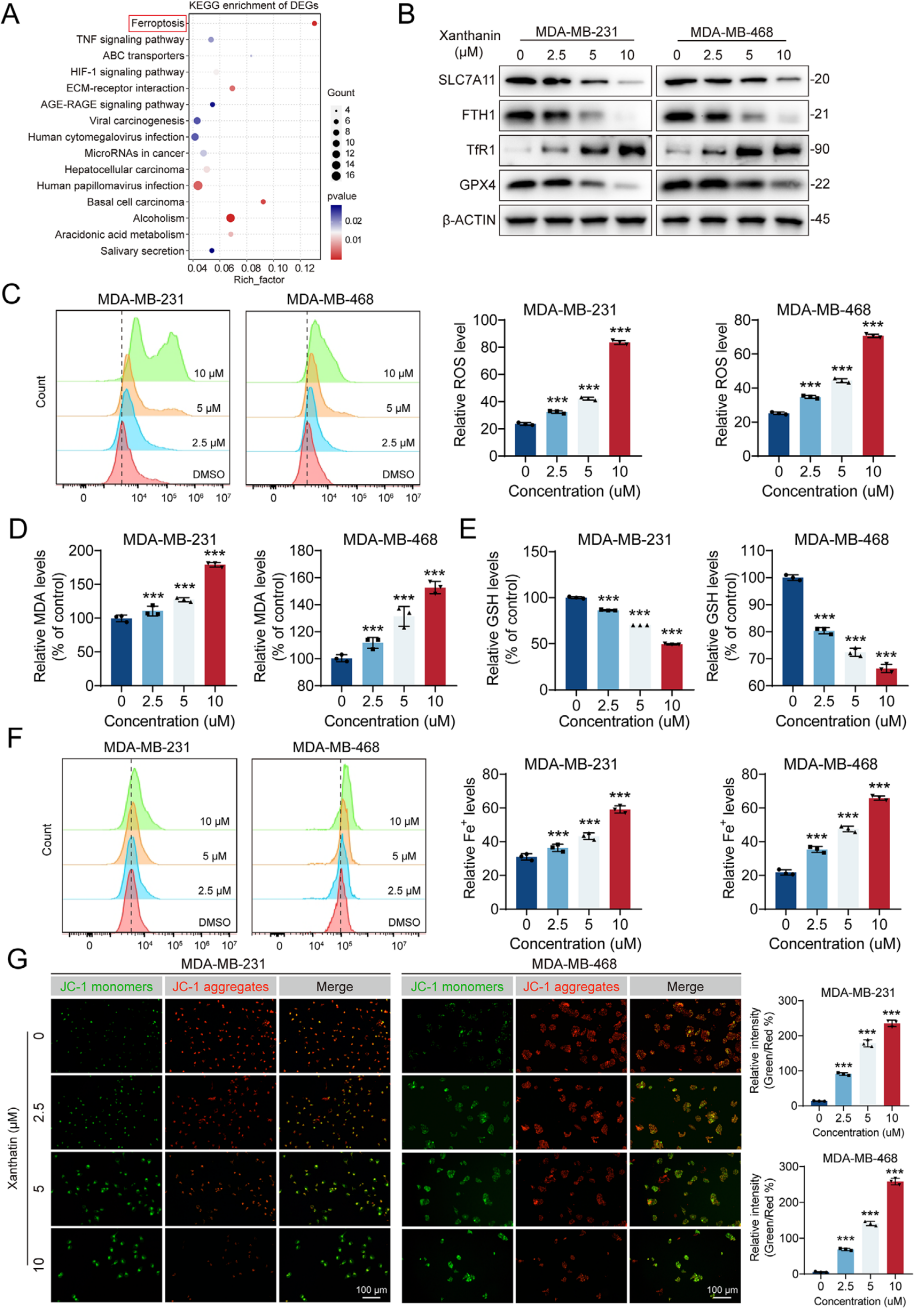
Xanthatin induces ferroptosis in TNBC cells. (A) KEGG enrichment analysis of DEGs in MDA‐MB‐231 cells treated with xanthatin for 48 h revealed significant enrichment in ferroptosis‐related pathways. (B) Western blot analysis showing the expression levels of ferroptosis‐associated proteins SLC7A11, FTH1, TfR1, and GPX4 in MDA‐MB‐231 and MDA‐MB‐468 cells after treatment with xanthatin for 48 h. (C) Flow cytometry analysis of ROS levels in MDA‐MB‐231 and MDA‐MB‐468 cells treated with xanthatin for 48 h. (D) MDA content in TNBC cells after xanthatin treatment for 48 h. (E) GSH levels in TNBC cells treated with xanthatin for 48 h. (F) Intracellular Fe^2^^+^ levels determined by flow cytometry in TNBC cells after xanthatin treatment for 48 h. (G) Representative images of JC‐1 staining showing loss of mitochondrial membrane potential in MDA‐MB‐231 and MDA‐MB‐468 cells after xanthatin treatment for 48 h. Scale bar = 100 µm. Bars, SDs; ^*^ 0.01< *p* < 0.05, ^**^ 0.001< *p* < 0.01, and ^***^ *p* < 0.001.

Given the potent growth‐inhibitory effect of xanthatin, we next sought to elucidate its underlying mechanism of action. Transcriptome sequencing of xanthatin‐treated TNBC cells identified 464 differentially expressed genes (DEGs), including 97 upregulated and 367 downregulated genes (Table S2). Subsequently, KEGG pathway enrichment analysis of these DEGs was performed to identify potential pharmacological targets (Figure 2A). Notably, the ferroptosis pathway was significantly enriched and ranked highest among the altered pathways, suggesting that ferroptosis may play a central role in xanthatin‐induced cytotoxicity. Ferroptosis is a distinct form of programmed cell death characterized by iron‐dependent lipid peroxidation [17]. Flow cytometry analysis demonstrated that xanthatin markedly increased intracellular ROS in a dose‐dependent manner (Figure 2C). Consistently, levels of lipid peroxidation products, including 4‐hydroxynonenal (4‐HNE) and malondialdehyde (MDA), were significantly elevated in xanthatin‐treated cells (Figure 2D; Figure S2A). Meanwhile, intracellular glutathione (GSH), the major antioxidant defense, was drastically depleted (Figure 2E). These results were corroborated at the protein level, where Western blot showed reduced expression of the cystine/glutamate antiporter xCT (SLC7A11) and glutathione peroxidase 4 (GPX4), both critical suppressors of ferroptosis (Figure 2B). Together, these findings indicate that xanthatin triggers severe oxidative stress and lipid peroxidation.

Given that ferroptosis execution relies on iron overload, we next assessed intracellular Fe^2^^+^ levels. Using a fluorescent Fe^2^^+^ probe, we observed a significant and dose‐dependent increase in free Fe^2^^+^ following xanthatin treatment (Figure 2F). Western blot analysis further revealed upregulation of transferrin receptor 1 (TfR1) and downregulation of ferritin heavy chain (FTH1), supporting the disruption of iron homeostasis (Figure 2B). Additionally, mitochondrial damage is a hallmark of ferroptosis. We visualized mitochondrial morphology using transmission electron microscopy (TEM). The results showed that mitochondria in xanthatin‐treated cells exhibited typical ferroptotic morphological characteristics, including shrinkage, increased membrane density, reduced or vanished cristae, and even rupture of the outer membrane (Figure S2B). Moreover, JC‐1 staining indicated a decrease in mitochondrial membrane potential, consistent with impaired mitochondrial energy metabolism (Figure 2G).

To further confirm the essential role of ferroptosis in xanthatin‐induced cell death, we employed three commonly used ferroptosis inhibitors: the lipid peroxidation blocker ferrostatin‐1 (Fer‐1), the iron chelator deferoxamine (DFO), and the ROS scavenger acetylcysteine (NAC). As expected, pretreatment with all three inhibitors markedly attenuated the suppressive effects of xanthatin on cell proliferation (Figure S2C,E), colony formation (Figure S2D) and cell survival (Figure S2F) capacity. This rescue experiment directly establishes ferroptosis as an indispensable mechanism underlying xanthatin's anticancer activity.

### 2.3 CISD1 is a Direct Binding Target of Xanthatin

**FIGURE 3:**
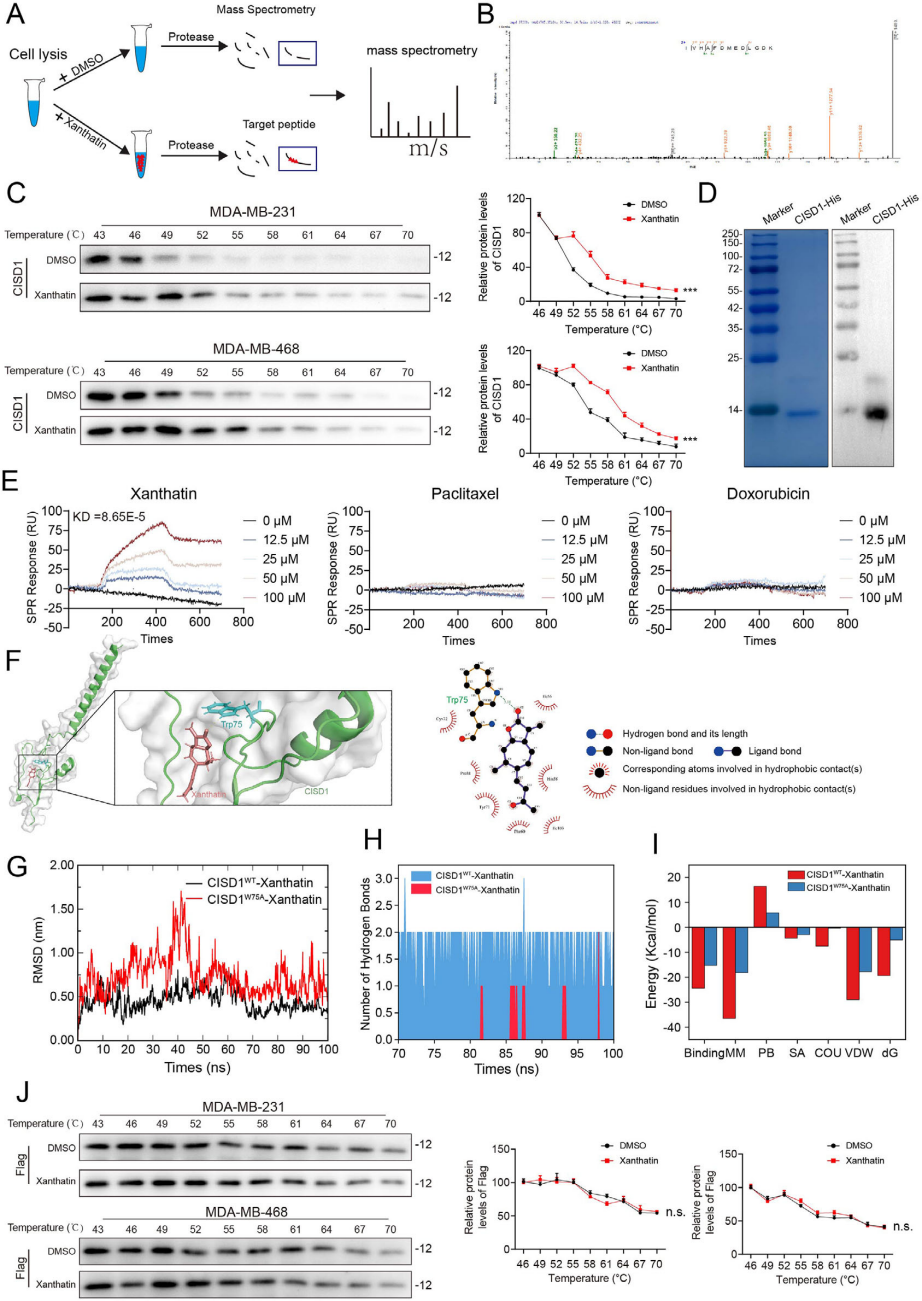
CISD1 is a direct binding target of Xanthatin. (A) Schematic of the DARTS‐MS assay. (B) The secondary mass spectrum of the CISD1 protein. (C) CETSA assay showing the thermal stability of CISD1 protein in MDA‐MB‐231 and MDA‐MB‐468 cells after treatment with xanthatin for 1 h. (D) SDS‐PAGE and Western blot showing recombinant CISD1‐His protein. (E) SPR analysis of the binding affinity of xanthatin, paclitaxel, and doxorubicin with CISD1‐His recombinant protein. (F) Molecular docking analysis showing the predicted binding mode of xanthatin with CISD1, and schematic diagram of the interaction between xanthatin and amino acid residues of CISD1. (G) RMSD trajectories of CISD1^WT–^xanthatin and CISD1^W75A–^xanthatin complexes during 100 ns MD simulations. (H) Time‐dependent hydrogen bond numbers between xanthatin and CISD1^WT^ or CISD1^W75A^. (I) Binding free energy decomposition of xanthatin with CISD1^WT^ and CISD1^W75A^, including Binding free energy (Binding), Molecular Mechanics energy (MM), Polar solvation energy (PB), Solvent accessible surface area energy (SA), Coulomb energy (COU), van der Waals energy (VDW), and total free energy (dG). (J) CETSA assay showing the thermal stability of exogenous CISD1‐Flag protein in MDA‐MB‐231 and MDA‐MB‐468 cells after treatment with xanthatin for 1 h. Bars, SDs; ^*^ 0.01< *p* < 0.05, ^**^ 0.001< *p* < 0.01, and ^***^ *p* < 0.001. n.s., no significance.

Precise identification of drug–protein interactions is essential for elucidating the pharmacological mechanisms of small molecules. To uncover the potential molecular targets of xanthatin, we employed the drug affinity responsive target stability and mass spectrometry (DARTS‐MS) assay. DARTS exploits the principle that drug binding protects target proteins from protease‐mediated degradation. Using this approach, we identified 86 differential proteins as candidate targets of xanthatin (Figure 3A; Table S3). Considering that xanthatin exerts its anti‐tumor activity through ferroptosis, we conducted a literature survey to examine the relevance of these candidates to ferroptosis and simultaneously performed molecular docking analysis to evaluate their binding potential. Among the 86 candidates, CISD1 ranked first and was therefore selected for experimental validation (Figure 3B; Table S3). Cellular thermal shift assay (CETSA) confirmed the direct binding of xanthatin to CISD1. Compared with the control, CISD1 in xanthatin‐treated cells exhibited enhanced thermal stability, indicating that the drug conferred resistance against heat‐induced denaturation (Figure 3C). To further validate this interaction, we performed surface plasmon resonance (SPR) analysis, which allows real‐time monitoring of molecular binding events. Purified CISD1‐His protein (Figure 3D) was immobilized onto a 3‐D photocrosslinked chip, and binding affinity was assessed with xanthatin, paclitaxel, and doxorubicin. Remarkably, only xanthatin displayed a specific interaction with CISD1, with a dissociation constant (KD) of 8.65 × 10^−^^8^, whereas paclitaxel and doxorubicin showed no detectable binding (Figure 3E).

Molecular docking results suggested that xanthatin might form a hydrogen bond with the tryptophan‐75 (Trp‐75) residue of CISD1 (Figure 3F). To further validate the structural basis of the xanthatin–CISD1 interaction, molecular dynamics (MD) simulations were conducted. The root mean square deviation (RMSD) analysis showed that the wild‐type CISD1 (CISD1^WT^)–xanthatin complex remained relatively stable throughout the 100 ns simulation, whereas the CISD1^W75A^ [Substitution of Trp‐75 (W) for alanine‐75 (A)] –xanthatin complex exhibited pronounced structural fluctuations, indicating impaired stability upon mutation (Figure 3G). Consistently, the root mean square fluctuation (RMSF) profile revealed increased flexibility in several regions of CISD1^W75A^, particularly around residues adjacent to the mutation site (Figure S3A). Hydrogen bond analysis further revealed persistent hydrogen bonding interactions in the CISD1^WT^ but CISD1^W75A^ (Figure 3H). Binding free energy calculations confirmed a significantly lower affinity for xanthatin toward CISD1^W75A^ compared with CISD1^WT^, with van der Waals and electrostatic interactions serving as the major contributors (Figure 3I). Residue energy contribution analysis highlighted Trp‐75 as the dominant hot spot, together with Tyr‐71, Leu‐101, and Ile‐56, underscoring the essential role of Trp‐75 in xanthatin binding (Figure S3B). Furthermore, we constructed a CISD1^W75A^‐Flag mutant plasmid and used CETSA to detect the binding of xanthatin to the exogenous mutant protein CISD1^W75A^‐Flag. As expected, xanthatin failed to increase the resistance of the CISD1^W75A^‐Flag mutant protein to heat denaturation (Figure 3J). To provide functional evidence that this interaction is required for the anticancer activity of xanthatin, we performed rescue experiments in a cellular context. We first established a stable CISD1‐knockdown cell line in TNBC cells, in which endogenous CISD1 was depleted with nearly 100% efficiency with shCISD1#1 plasmid (Figure S3C). In this background, we rescued the expression by transfecting either CISD1^WT^ plasmid or the CISD1^W75A^ plasmid. Subsequent functional assays demonstrated that xanthatin treatment significantly inhibited cell viability (Figure S3D), suppressed colony formation (Figure S3E), and elevated intracellular Fe^2^^+^ levels (Figure S3F) in cells rescued with CISD1^WT^. In stark contrast, these cytotoxic and ferroptosis‐promoting effects of xanthatin were markedly attenuated in cells rescued with CISD1^W75A^. Collectively, these results provide strong evidence that CISD1 is a direct binding target of xanthatin, and that the Trp‐75 residue plays a pivotal role in mediating this interaction.

### 2.4 Xanthatin Targets CISD1 to Induce Mitochondrial Autophagy

**FIGURE 4:**
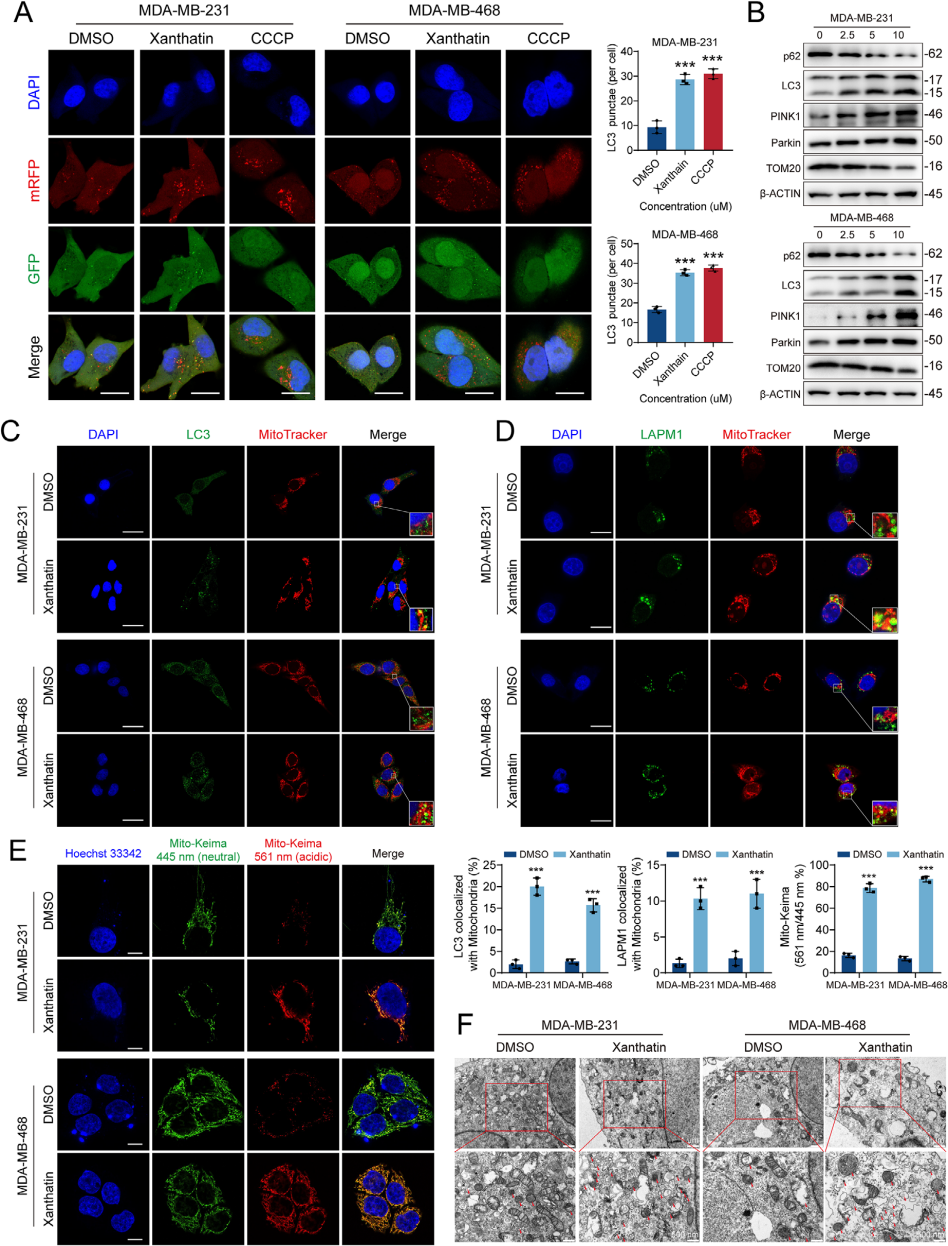
Xanthatin targets CISD1 to induce mitochondrial autophagy. (A) mRFP‐GFP‐LC3 reporter assay showing autophagic flux in MDA‐MB‐231 and MDA‐MB‐468 cells treated with xanthatin or 10 µm CCCP. Representative images display GFP (green), mRFP (red), and merged puncta. Scale bar = 20 µm. (B) Western blot analysis of autophagy‐ and mitophagy‐related proteins p62, LC3, PINK1, Parkin, and TOM20 in MDA‐MB‐231 and MDA‐MB‐468 cells after treatment with xanthatin for 48 h. (C) Immunofluorescence staining of LC3 and MitoTracker in MDA‐MB‐231 and MDA‐MB‐468 cells treated with xanthatin for 48 h. Scale bar = 20 µm. (D) Immunofluorescence staining of LAMP1 and MitoTracker in MDA‐MB‐231 and MDA‐MB‐468 cells treated with xanthatin for 48 h. Scale bar = 20 µm. (E) Representative mito‐Keima images showing increased acidic mitochondrial signals in MDA‐MB‐231 and MDA‐MB‐468 cells after xanthatin treatment for 48 h. Scale bar = 10 µm. (F) TEM images of MDA‐MB‐231 and MDA‐MB‐468 cells treated with xanthatin for 48 h. autophagic vacuoles are indicated with red arrows. Scale bar = 1 µm (upper panels), 500 nm (lower panels). Bars, SDs; ^*^ 0.01< *p* < 0.05, ^**^ 0.001< *p* < 0.01, and ^***^ *p* < 0.001.

Intriguingly, accumulating evidence suggests that CISD1 dysfunction not only accelerates ferroptosis but also contributes to mitochondrial damage [12, 18]. Damaged mitochondria are often selectively eliminated via mitophagy, serving as a protective mechanism to maintain cellular homeostasis. Based on this rationale, we hypothesized that xanthatin treatment may activate mitophagy in TNBC cells. To test this hypothesis, MDA‐MB‐231 and MDA‐MB‐468 cells were transfected with the mRFP‐GFP‐LC3 dual‐fluorescent reporter, which distinguishes autophagosomes (yellow puncta, GFP^+^RFP^+^) from autolysosomes (red puncta, GFP^−^RFP^+^) due to GFP quenching in acidic compartments. Confocal imaging revealed a marked increase in both yellow and red puncta following xanthatin or classical mitophagy inducer carbonyl cyanide 3‐chlorophenylhydrazone (CCCP) exposure, suggesting enhanced autophagic flux (Figure 4A). Consistently, Western blot analysis showed a dose‐dependent decrease in TOM20 and p62, alongside elevated levels of LC3‐II, PINK1, and Parkin (Figure 4B). Since PINK1 accumulation and Parkin recruitment are canonical hallmarks of mitophagy initiation during mitochondrial depolarization, these results strongly indicate activation of PINK1/Parkin‐mediated mitophagy. Further evidence was provided by immunofluorescence colocalization assays. LC3 exhibited strong colocalization with mitochondria labeled by Mito‐Tracker (Figure 4C), and mitochondria showed increased colocalization with the lysosomal marker LAMP1 (Figure 4D), confirming their delivery into degradative autolysosomes. Furthermore, we employed the mitochondria‐targeted pH‐sensitive reporter Mito‐Keima. Unlike conventional autophagy markers, Mito‐Keima enables direct measurement of mitochondrial delivery to acidic lysosomes based on excitation ratio changes. Live‐cell confocal imaging showed that xanthatin treatment markedly increased Mito‐Keima signals preferentially excited at 561 nm relative to 445 nm in both MDA‐MB‐231 and MDA‐MB‐468 cells, indicating enhanced mitochondrial localization within acidic compartments (Figure 4E). Consistent with these findings, TEM offered ultrastructural validation, revealing characteristic double‐membrane autophagosomes encapsulating fragmented mitochondria in xanthatin‐treated cells, whereas such structures were rare in control cells (Figure 4F).

Given that autophagy is a multi‐step dynamic process, increased LC3 puncta may result either from enhanced initiation or impaired degradation. To distinguish between these possibilities, we employed the late‐stage autophagy inhibitor chloroquine (CQ) and the early‐stage inhibitor 3‐methyladenine (3‐MA). CQ blocks the fusion of autophagosomes with lysosomes, thereby preventing degradation. If xanthatin promotes autophagy initiation, the presence of CQ should lead to further accumulation of LC3 puncta, as newly formed autophagosomes cannot be degraded. In contrast, 3‐MA inhibits class III PI3K activity, thereby blocking autophagy at an early stage. The result showed that CQ co‐treatment with xanthatin markedly increased both LC3 puncta and protein levels compared with xanthatin alone, whereas 3‐MA pretreatment significantly attenuated xanthatin‐induced LC3 accumulation (Figure S4A,B). Together, these findings demonstrate that xanthatin‐induced LC3 upregulation results from activation of autophagy initiation rather than blockade of autophagic degradation.

### 2.5 Xanthatin‐Induced Mitophagy is Activated in Response to Ferroptotic Stress

**FIGURE 5:**
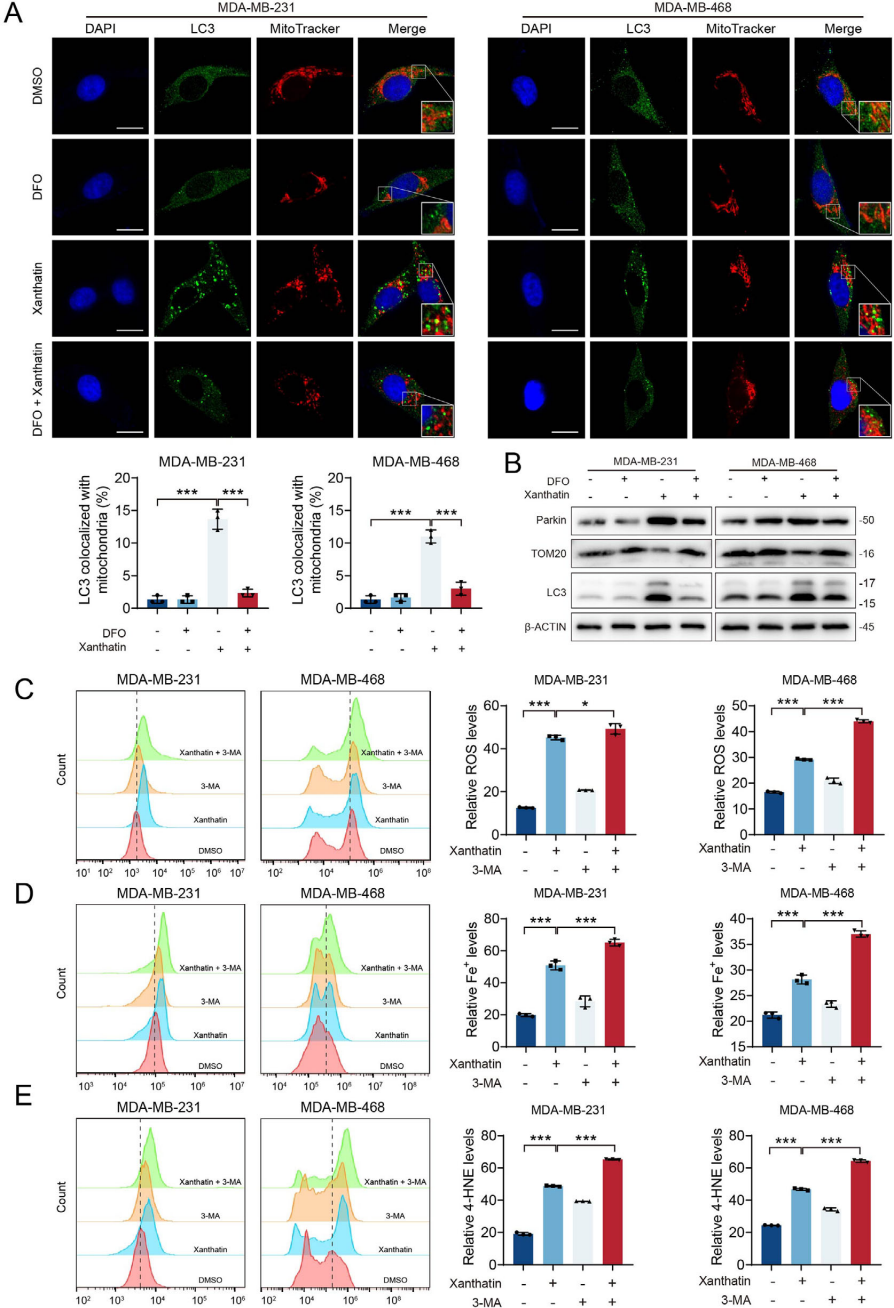
Xanthatin‐induced mitophagy is activated in response to ferroptotic stress. (A) Immunofluorescence staining showing colocalization of LC3 and mitochondria in MDA‐MB‐231 and MDA‐MB‐468 cells treated with xanthatin for 48 h, with or without DFO pretreatment (20 µm, 12 h). Scale bar = 20 µm. (B) Western blot analysis of mitophagy‐related proteins Parkin, TOM20, and LC3 in MDA‐MB‐231 and MDA‐MB‐468 cells treated with xanthatin in the presence or absence of 20 µM DFO. (C‐E) Flow cytometric analysis of intracellular ROS, Fe^2^^+^ and 4‐HNE levels in MDA‐MB‐231 and MDA‐MB‐468 cells treated with xanthatin for 48 h, with or without 1 mM 3‐MA pretreatment.

To define the functional interplay between ferroptosis and mitophagy following xanthatin treatment, we pharmacologically modulated iron availability and autophagy initiation. Given that CISD1 is a key regulator of mitochondrial iron homeostasis, we first examined whether iron dysregulation contributes to xanthatin‐induced mitophagy by using the iron chelator deferoxamine B (DFO). As shown in Figure 5A,B, DFO markedly reduced LC3 colocalization with mitochondria in both MDA‐MB‐231 and MDA‐MB‐468 cells treated with xanthatin, indicating diminished mitophagy (Figure 5A). Consistently, DFO largely reversed the xanthatin‐induced accumulation of Parkin and LC3‐II and restored the mitochondrial protein TOM20 (Figure 5B). We next investigated how autophagy influences ferroptotic stress by inhibiting autophagy initiation with 3‐MA. Flow cytometric analysis revealed that autophagy inhibition substantially enhanced xanthatin‐induced ROS accumulation in both TNBC cell lines (Figure 5C). In parallel, intracellular Fe^2^^+^ and 4‐HNE levels were further elevated upon combined treatment with xanthatin and 3‐MA compared with xanthatin alone (Figure 5D,E). Taken together, these results demonstrate that xanthatin induces iron‐dependent ferroptotic stress, while mitophagy is activated as a subsequent response to this stress. Suppression of iron accumulation attenuates mitophagy, whereas inhibition of autophagy amplifies ferroptosis, highlighting a ferroptosis‐dominant response pattern upon xanthatin treatment.

To further validate the generality of xanthatin‐induced ferroptosis and mitophagy in TNBC cells, we extended our analyses to an additional TNBC cell line, BT‐549. Cell viability assays revealed that xanthatin suppressed BT‐549 cell proliferation in a dose‐dependent manner (Figure S5A), which was further confirmed by a marked reduction in clonogenic survival (Figure S5B). Consistent with ferroptotic features observed in MD‐MB‐231 and MDA‐MB‐468 cells, xanthatin treatment led to a significant increase in intracellular ROS levels and Fe^2^^+^ accumulation in BT‐549 cells (Figure S5C,D). We next assessed whether xanthatin also induces mitophagy in BT‐549 cells. Immunofluorescence analysis showed enhanced colocalization of LC3 puncta with mitochondria upon xanthatin treatment, indicating activation of mitochondrial autophagy (Figure S5E). In parallel, Western blot analysis demonstrated a dose‐dependent increase in LC3‐II, PINK1, and Parkin levels, accompanied by a reduction in the mitochondrial protein TOM20 (Figure S5F), further supporting the induction of mitophagy. Collectively, these results demonstrate that xanthatin consistently induces ferroptotic stress and a mitophagic response across multiple TNBC cell lines.

### 2.6 CISD1 is Highly Expressed in TNBC and Represents a Potential Therapeutic Target

**FIGURE 6:**
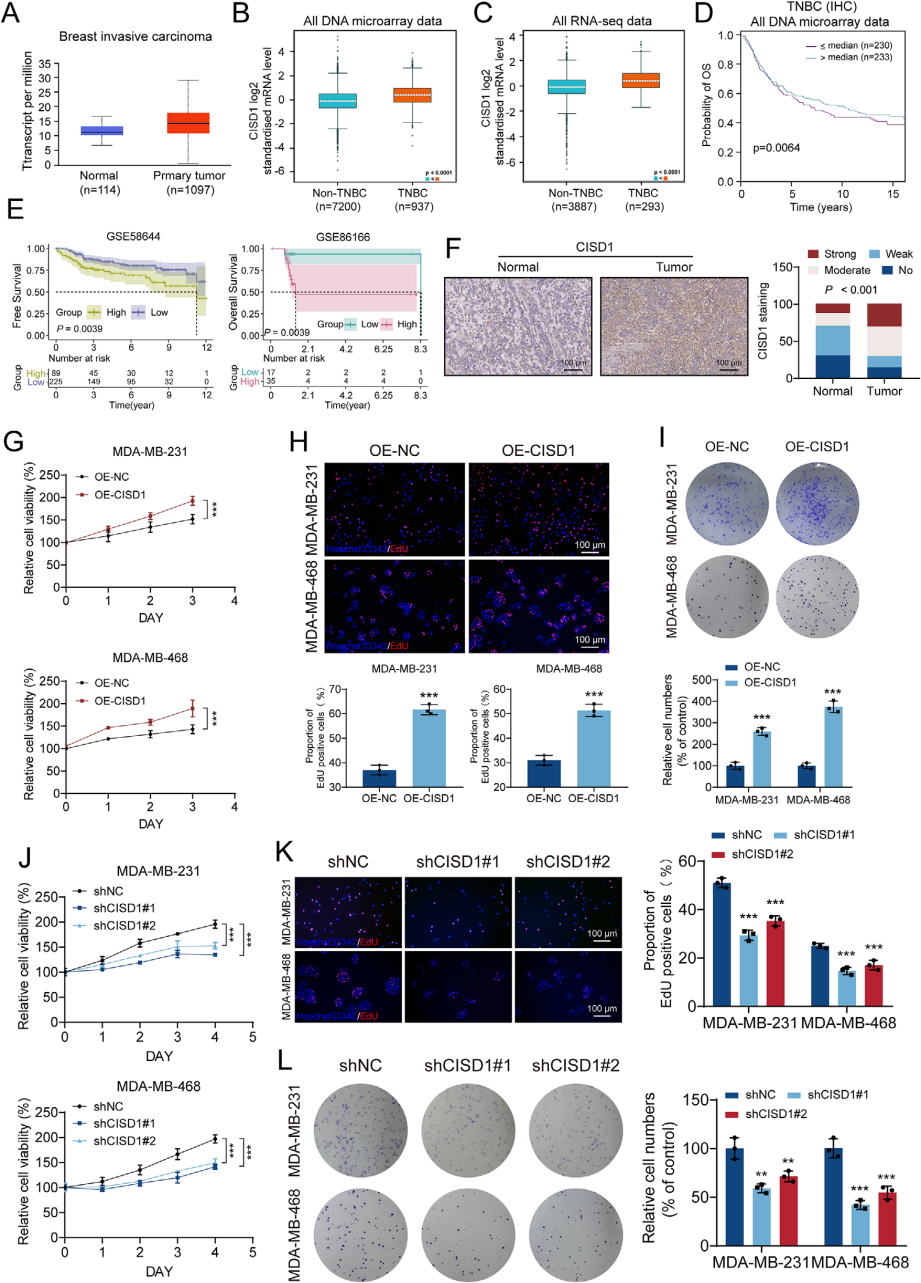
CISD1 expression is upregulated in TNBC and represents a potential therapeutic target. (A‐C) The expression of CISD1 in normal and tumor tissues were analyzed through UALCAN website (https://ualcan.path.uab.edu/index.html) and BCa Gene‐Expression Miner website (http://bcgenex.ico.unicancer.fr/BC‐GEM/GEM‐Accueil.php?js=1). (D) Kaplan‐Meier survival curve analysis indicated that high CISD1 expression was associated with poor prognosis in TNBC patients. (E) Kaplan–Meier analysis of recurrence‐free survival (GSE58644) and overall survival (GSE86166) stratified by high or low CISD1 expression. (F) Representative images and expression pattern of CISD1 in 48 TNBC tussues and adjacent normal tissues. (G–I) CCK‐8 (G), EdU proliferation (H) and colony formation assay performed in OE‐NC and OE‐CISD1 stable cell lines. Scale bar for EdU = 100 µm. (J–L) Proliferation assays including CCK‐8 (J) and EdU incorporation (K) and colony formation assay (L) performed in shNC and shCISD1 stable knockdown cell lines. Scale bar for EdU = 100 µm. Bars, SDs; ^*^ 0.01< *p* < 0.05, ^**^ 0.001< *p* < 0.01, and ^***^ *p* < 0.001.

CISD1 has been implicated in multiple oncogenic processes, including non‐small cell lung cancer, [14] gastric cancer, [19] and breast cancer [13]. Although previous studies suggested that CISD1 may serve as a prognostic biomarker in TNBC, its functional role remains largely undefined. To investigate this, we began with bioinformatic database analysis. Analysis using the UALCAN platform based on TCGA data showed that CISD1 expression was significantly upregulated in invasive breast carcinoma compared with normal tissues (Figure 6A). Further analysis using bc‐GenExMiner v5.0, a dedicated breast cancer transcriptome tool, consistently revealed that CISD1 expression was markedly higher in TNBC than in non‐TNBC subtypes (Figure 6B,C). Survival analysis indicated that high CISD1 expression was significantly associated with poor patient prognosis (Figure 6D), a conclusion further supported by survival analysis of two independent GEO datasets (Figure 6E). To confirm this phenomenon at the tissue protein level, we performed immunohistochemistry on 48 pairs of TNBC tumor tissues and adjacent normal tissues. Tissue microarray analysis showed that the proportion of CISD1‐high expression was significantly greater in tumor tissues (71%, 34/48) than in adjacent tissues (30%, 14/48) (Figure 6F). Subsequently, we validated these findings at the cellular level. qPCR and Western blot assays confirmed that CISD1 expression was significantly elevated in TNBC cell lines compared with MCF10A (Figure S6A,B).

To further elucidate the biological function of CISD1 in TNBC, we generated stable cell lines with either CISD1 overexpression (OE‐CISD1) or knockdown (shCISD1#1 and shCISD1#2) via lentiviral transduction, followed by puromycin selection. Expression changes were validated by qPCR and Western blot (Figures S3C and S6C,D). Functional assays revealed that CISD1 overexpression significantly enhanced TNBC cell proliferation and colony‐forming ability (Figure 6G–I), whereas CISD1 silencing produced the opposite effect, strongly suppressing these malignant phenotypes (Figure 6J–L). Taken together, these findings demonstrate that CISD1 is aberrantly overexpressed in TNBC, correlates with poor prognosis, and exerts a tumor‐promoting role by enhancing proliferative capacity, supporting its potential as a therapeutic target in TNBC.

### 2.7 The Anticancer Activity of Xanthatin Relies on CISD1

**FIGURE 7:**
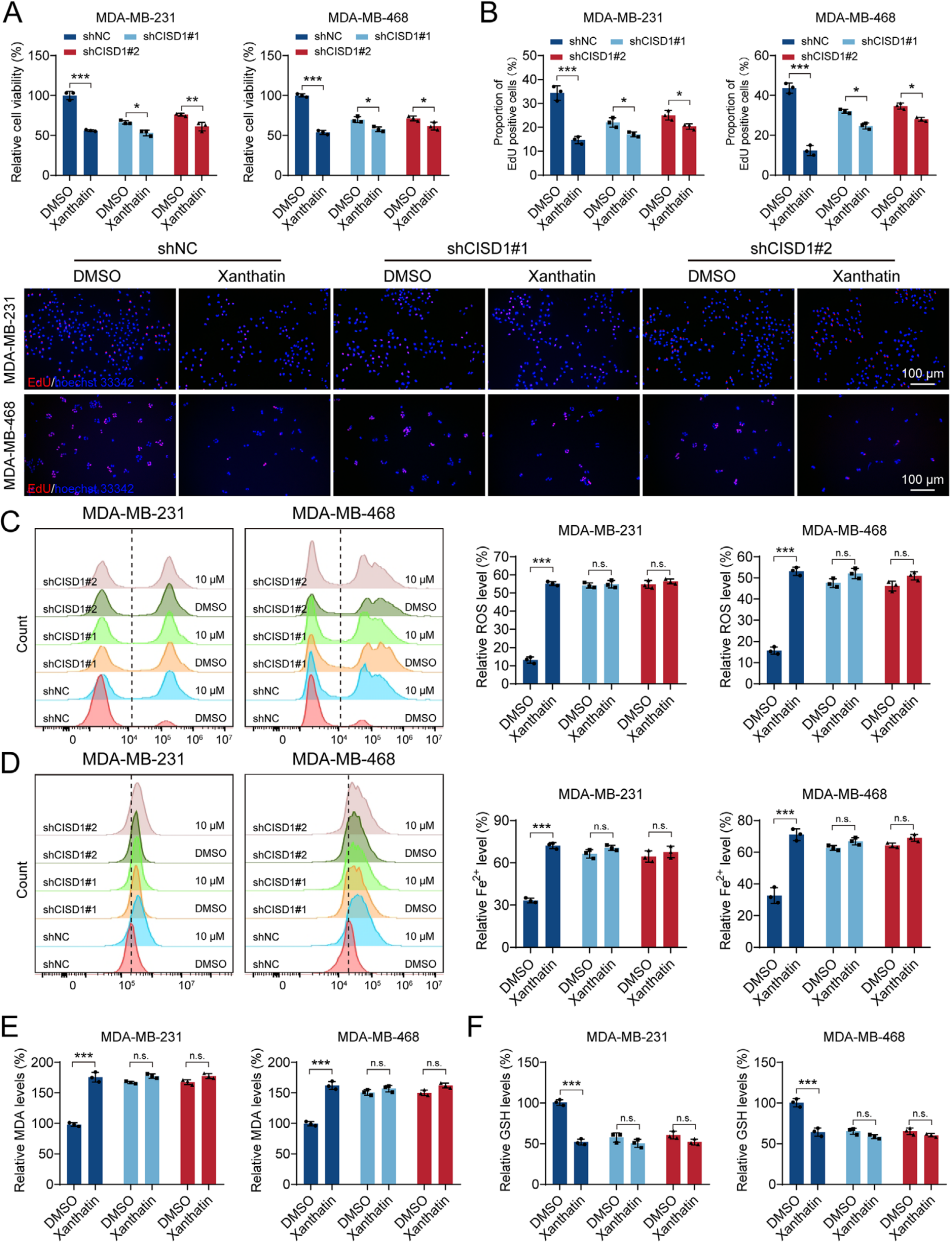
The anticancer activity of xanthatin relies on CISD1. (A‐B) CCK‐8 assay (A) and EdU incorporation assay (B) in MDA‐MB‐231 and MDA‐MB‐468 stable knockdown cell lines (shCISD1#1, shCISD1#2) and control cells (shNC) treated with DMSO or xanthatin for 48 h. Scale bar for EdU = 100 µm. (C‐D) Flow cytometry analysis of ROS levels (C) and intracellular Fe^2^^+^ levels (D) in shNC, shCISD1#1, and shCISD1#2 stable knockdown cell lines treated with DMSO or xanthatin for 48 h. (E‐F) Biochemical detection of MDA levels (E) and GSH levels (F) in shNC, shCISD1#1, and shCISD1#2 stable knockdown cell lines treated with DMSO or xanthatin for 48 h. Bars, SDs; ^*^ 0.01< *p* < 0.05, ^**^ 0.001< *p* < 0.01, and ^***^ *p* < 0.001. n.s., no significance.

To determine whether the anti‐tumor activity of xanthatin relies on CISD1, we next examined the effects of xanthatin in CISD1‐silenced TNBC cells. CCK‐8 and EdU assays demonstrated that CISD1 knockdown significantly attenuated the growth‐inhibitory effect of xanthatin (Figure 7A,B), indicating that the anti‐proliferative activity of xanthatin is largely CISD1‐dependent. We then evaluated ferroptosis‐related markers. Compared with shNC controls, CISD1 knockdown alone led to elevated intracellular ROS and Fe^2^^+^ levels, consistent with impaired mitochondrial homeostasis. However, the ability of xanthatin to further increase these markers was markedly reduced or abolished in CISD1‐deficient cells (Figure 7C,D). Similarly, xanthatin‐induced lipid peroxidation, reflected by MDA accumulation, and GSH depletion were eliminated in the context of CISD1 knockdown (Figure 7E,F). Autophagic responses showed a parallel pattern. In shNC cells, xanthatin robustly promoted LC3 lipidation and puncta formation, consistent with enhanced autophagy. In contrast, this effect was completely abrogated in CISD1 knockdown cells (Figure S7A). Collectively, these results provide compelling evidence that xanthatin‐induced ferroptosis and mitophagy are strictly dependent on the presence of CISD1. Without CISD1, xanthatin fails to trigger oxidative stress, lipid peroxidation, or autophagic activation, underscoring CISD1 as the critical mediator of xanthatin's anti‐cancer activity.

### 2.8 Xanthatin Induces CISD1 Degradation Through Parkin‐mediated Ubiquitination

**FIGURE 8:**
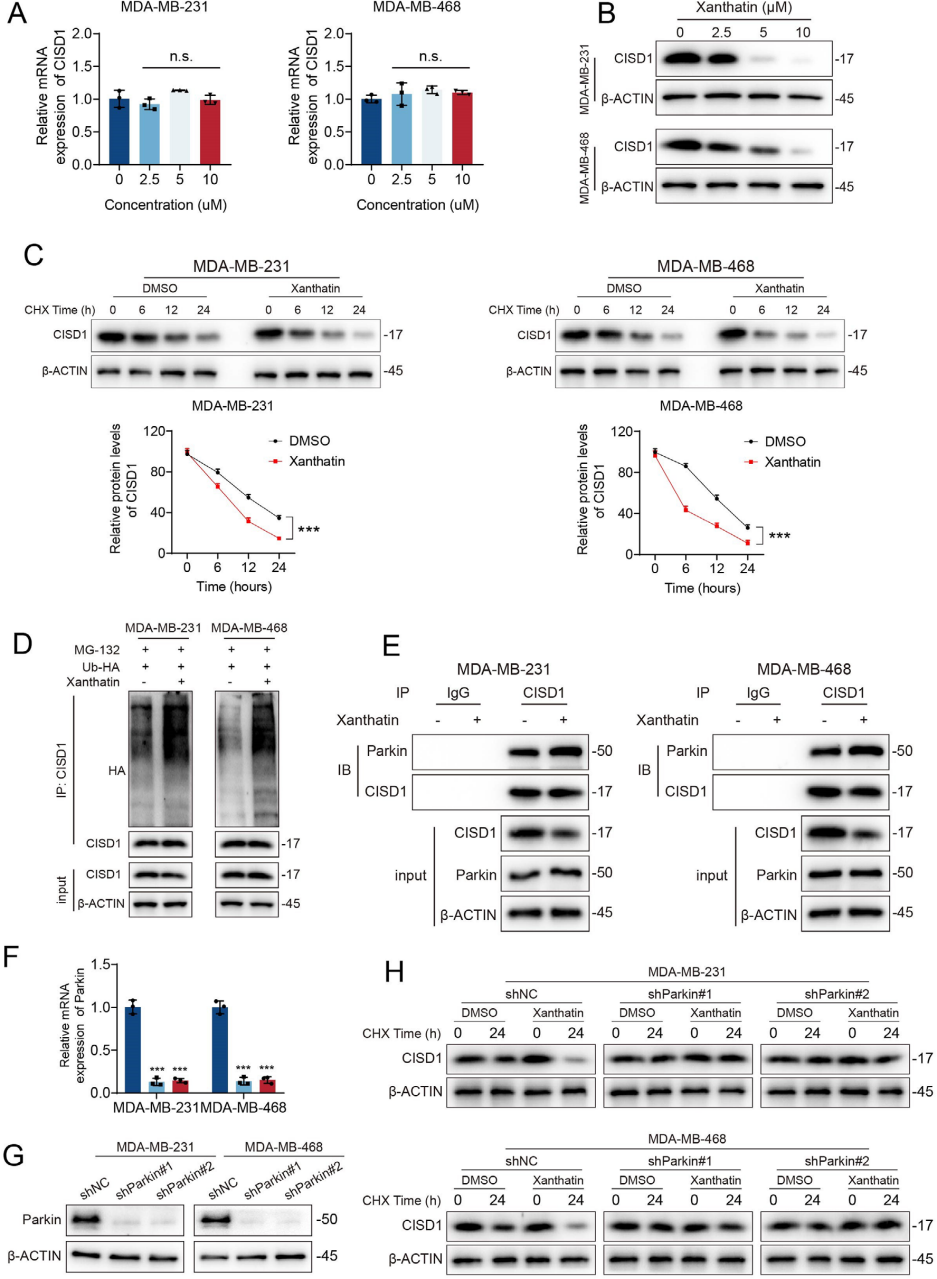
Xanthatin induces CISD1 degradation through Parkin‐mediated ubiquitination. (A, B) CISD1 mRNA and protein levels in MDA‐MB‐231 and MDA‐MB‐468 cells treated with increasing concentrations of xanthatin for 48 h were determined by qPCR (A) and Western blot (B). (C) Western blot analysis of CISD1 expression in MDA‐MB‐231 and MDA‐MB‐468 cells treated with 100 µg/mL CHX in the presence or absence of xanthatin at the indicated time points. (D) After transfection with Ub‐HA plasmid for 36 h, cells were exposed to 5 µm xanthatin together with 5 µm MG‐132 for 24 h. Immunoprecipitation with anti‐CISD1 antibody was then performed, and ubiquitinated CISD1 was detected using anti‐HA antibody. (E) Co‐immunoprecipitation analysis of the interaction between CISD1 and Parkin in cells treated with DMSO or xanthatin for 48 h. (F,G) qPCR (F) and Western blot (G) analysis of Parkin expression in MDA‐MB‐231 and MDA‐MB‐468 stable knockdown cell lines. (H) Western blot analysis of CISD1 protein stability in shNC, shParkin#1, and shParkin#2 cells treated with 100 µg/mL CHX in the presence or absence of xanthatin for the 0 or 12 h. Bars, SDs; ^*^ 0.01< *p* < 0.05, ^**^ 0.001< *p* < 0.01, and ^***^ *p* < 0.001.

To further elucidate the molecular mechanism by which xanthatin regulates CISD1, we first examined its effects on CISD1 transcriptional and translational levels. Results showed that xanthatin treatment did not significantly alter CISD1 mRNA expression (Figure 8A), but dose‐dependently reduced its protein level (Figure 8B), suggesting post‐translational regulation of CISD1 stability by the xanthatin. To test this hypothesis, we employed the protein synthesis inhibitor cycloheximide (CHX) to block de novo protein synthesis and monitored CISD1 degradation kinetics. The results demonstrated that xanthatin significantly shortened the half‐life of CISD1 protein (Figure 8C), confirming drug‐accelerated degradation.

Given previous reports that CISD1 undergoes Parkin‐mediated ubiquitination for degradation [18, 20], coupled with our observation of PINK1/Parkin pathway activation, we further investigated the involvement of the ubiquitin‐proteasome system. Western blot analysis revealed that xanthatin markedly enhanced ubiquitin conjugation to CISD1 (Figure 8D), indicating drug‐triggered ubiquitin labeling. Co‐immunoprecipitation assays further verified that xanthatin treatment significantly promoted the interaction between Parkin and CISD1 (Figure 8E). To confirm the necessity of Parkin in this process, we established a Parkin‐stable knockdown cell line. qPCR and Western blot results confirmed the efficient knockdown of Parkin at both mRNA and protein levels, respectively (Figure 8F,G). Crucially, in Parkin‐knockdown (shParkin) cells, xanthatin treatment no longer reduced CISD1 protein levels (Figure 8H). Collectively, these results demonstrate that xanthatin recruits the E3 ubiquitin ligase Parkin to enhance CISD1 ubiquitination, thereby driving its proteasomal degradation in a manner strictly dependent on Parkin.

### 2.9 Xanthatin Suppresses Orthotopic TNBC Tumor Growth In Vivo Without Systemic Toxicity

**FIGURE 9:**
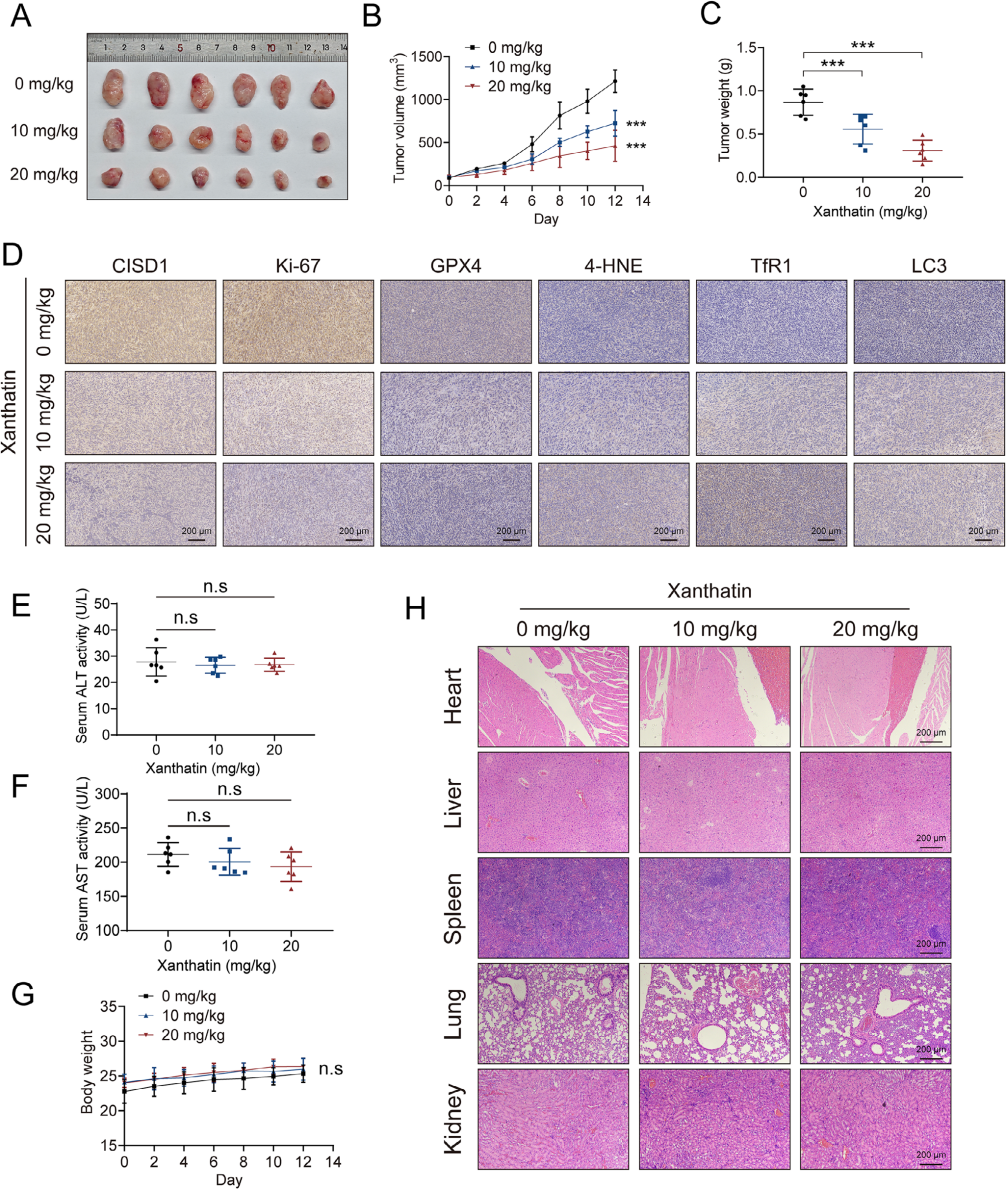
Xanthatin suppresses orthotopic TNBC tumor growth in vivo without systemic toxicity. (A) Representative images of orthotopic breast tumors excised from BALB/c mice. (B) Tumor growth curves monitored every two days throughout the treatment period. (C) Final tumor weights measured at the end of the experiment. (D) Representative immunohistochemical staining images of CISD1, Ki‐67, GPX4, 4‐HNE, TfR1, and LC3 in tumor tissues from mice treated with vehicle or xanthatin (10 or 20 mg/kg). Scale bar = 200 µm. (E–G) Serum ALT level (E), AST level (F), and body weight (G) were recorded after treatment. (H) Representative H&E staining images of major organs (heart, liver, spleen, lung, and kidney) collected from mice treated with different doses of xanthatin. Scale bar = 200 µm. Bars, SDs; ^*^ 0.01< *p* < 0.05, ^**^ 0.001< *p* < 0.01, and ^***^ *p* < 0.001. n.s., no significance.

To evaluate the in vivo anti‐tumor efficacy and safety of xanthatin, we established an orthotopic TNBC model in BALB/c mice using 4T1 cells. Mice were randomized into three groups and treated with vehicle, 10 mg/kg xanthatin, or 20 mg/kg xanthatin via intraperitoneal injection every two days. Tumor growth was monitored during treatment. The results revealed that xanthatin administration significantly reduced tumor volume and growth rate in a dose‐dependent manner (Figure 9A,B). Correspondingly, final tumor weights were significantly lower in the treatment groups compared to controls (Figure 9C). Further immunohistochemical analysis of tumor tissues revealed that xanthatin treatment not only significantly downregulated the expression of its direct target CISD1 but also concurrently reduced the level of the cell proliferation marker Ki‐67, providing molecular corroboration for the observed suppression of tumor growth. Notably the altered expression of ferroptosis‐related markers (decreased GPX4, increased 4‐HNE and TfR1) and the autophagy marker LC3 consistently indicated that xanthatin effectively activated both ferroptosis and autophagy pathways in vivo (Figure 9D). This aligns with the cell death mechanisms it induces at the cellular level and collectively underpins its in vivo anti‐tumor efficacy. To assess systemic toxicity, serum biochemical parameters were examined. No significant differences in alanine aminotransferase (ALT) or aspartate aminotransferase (AST) levels were observed (Figure 9E,F), indicating no evident hepatotoxicity. Moreover, body weight remained stable across all groups throughout the experiment (Figure 9G). Histopathological analysis of major organs (heart, liver, spleen, lung, and kidney) showed no overt structural abnormalities or pathological lesions following xanthatin treatment (Figure 8H). Together, these findings demonstrate that xanthatin exerts potent anti‐tumor activity in vivo by suppressing TNBC progression, while exhibiting minimal systemic toxicity, supporting its translational potential as a therapeutic candidate.

## 3 Discussion

TNBC is a highly aggressive subtype with limited therapeutic options. In this study, we identified the natural compound xanthatin as a potent inhibitor of TNBC growth. Mechanistic investigations revealed that xanthatin directly targets the mitochondrial protein CISD1, leading to its destabilization. This disruption induces ferroptosis and activates PINK1/Parkin‐mediated mitophagy, together amplifying mitochondrial stress and suppressing tumor survival. Furthermore, xanthatin displayed strong antitumor efficacy in orthotopic TNBC models with minimal systemic toxicity.

Natural compounds have historically provided a rich source for anticancer drug discovery, with many clinically approved chemotherapeutics, such as paclitaxel and camptothecin derivatives, originating from plants. Their structural diversity and ability to modulate multiple molecular targets make them particularly valuable for treating complex malignancies like TNBC, where single‐target therapies often fail [21, 22]. Among these compounds, xanthatin, a sesquiterpene lactone isolated from Xanthium species, has recently gained attention for its antitumor potential. Previous studies have demonstrated that xanthatin suppresses proliferation and induces apoptosis in various cancers, including non‐small cell lung cancer, [23] hepatocellular carcinoma, [24] and gastric cancer [25]. These findings suggest that xanthatin exerts broad‐spectrum anticancer activity through diverse mechanisms. Our study expands this knowledge by showing, for the first time, that xanthatin has strong inhibitory effects against TNBC cells, while exerting negligible toxicity on normal mammary epithelial cells. Importantly, unlike conventional chemotherapeutic agents that mainly act by inducing apoptosis, xanthatin was found to simultaneously trigger ferroptosis and mitophagy, two regulated cell death processes intimately linked to mitochondrial homeostasis. This dual mechanism not only distinguishes xanthatin from existing therapies but also provides a potential strategy to overcome drug resistance, a major clinical obstacle in TNBC treatment. Furthermore, its natural origin and relatively low toxicity profile make xanthatin a promising scaffold for further drug development. Collectively, these findings underscore the continuing importance of natural products in cancer therapeutics and highlight xanthatin as a unique compound with translational potential for TNBC.

A growing body of evidence has recognized CISD1 (mitoNEET) as a critical mitochondrial regulator that links iron metabolism, oxidative stress, and cancer progression [26, 27]. Consistent with previous reports that CISD1 is upregulated in multiple solid tumors and predicts unfavorable prognosis, our bioinformatic analyses and experimental validation demonstrated that CISD1 is highly expressed in TNBC and associated with reduced patient survival. Functionally, our gain‐ and loss‐of‐function assays provided direct evidence that CISD1 promotes TNBC proliferation: overexpression significantly enhanced cell growth and colony formation, whereas shRNA‐mediated knockdown produced the opposite effect. These observations confirm CISD1 as a bona fide oncogenic driver in TNBC. It is worth noting that while we detected elevated CISD1 expression in TNBC cells compared to normal mammary epithelial cells, the present study, by design, focused on elucidating the mechanism within the TNBC context. Whether CISD1 overexpression and the resultant vulnerability to its inhibition are specific to TNBC relative to other breast cancer subtypes remains an open question that warrants systematic investigation in future studies. What distinguishes our work from earlier studies is the mechanistic link we established between CISD1 and xanthatin. While CISD1 has been recognized as a negative regulator of ferroptosis, our results demonstrated that pharmacological destabilization of CISD1 by xanthatin disrupts this protective function. Specifically, xanthatin treatment accelerated CISD1 protein degradation and consequently amplified iron accumulation, ROS elevation, and lipid peroxidation, as well as activated PINK1/Parkin‐mediated mitophagy. Importantly, these effects were abolished in CISD1‐knockdown cells, where xanthatin failed to induce ferroptotic and autophagic responses, thereby confirming CISD1 as an indispensable mediator of xanthatin's anticancer activity. From a translational perspective, these findings provide two important implications. First, CISD1 could serve as a biomarker to stratify TNBC patients who may benefit from ferroptosis‐inducing therapies. Second, small molecules such as xanthatin, which directly bind and destabilize CISD1, provide proof‐of‐concept for targeting mitochondrial vulnerabilities in cancer. Given the persistent challenge of therapeutic resistance in TNBC, CISD1 inhibition may represent an innovative approach to sensitize tumors to ferroptosis while limiting systemic toxicity.

Our mechanistic investigations revealed that xanthatin exerts its anticancer activity primarily through destabilization of CISD1, which serves as a key mitochondrial regulator of iron homeostasis. Pharmacological degradation of CISD1 disrupted mitochondrial iron balance, resulting in elevated ferrous iron, increased ROS production, and accumulation of lipid peroxidation products, thereby driving ferroptosis. This finding is consistent with earlier studies implicating CISD1 as a negative regulator of ferroptosis, but extends the concept by demonstrating that direct small‐molecule targeting of CISD1 is feasible and effective in TNBC cells. Interestingly, we also observed that xanthatin‐mediated CISD1 depletion caused profound mitochondrial damage, including collapse of the cristae and loss of membrane potential. These alterations activated the PINK1/Parkin pathway, which recruited Parkin to depolarized mitochondria and promoted ubiquitin‐mediated mitophagy. Thus, xanthatin treatment not only induced ferroptosis but also triggered selective clearance of damaged mitochondria. The coexistence of ferroptosis and mitophagy in our system underscores a dual‐attack effect. On one hand, ferroptosis directly compromises cell viability through iron‐dependent lipid peroxidation. On the other hand, mitophagy contributes to metabolic stress by depleting dysfunctional mitochondria and amplifying energy failure. Rather than functioning independently, these processes appear to reinforce each other: ferroptosis‐derived oxidative stress aggravates mitochondrial injury, while mitophagy‐driven organelle turnover enhances vulnerability to iron dysregulation. Together, this interplay explains the potent anticancer activity of xanthatin and suggests that simultaneous activation of ferroptosis and mitophagy represents a promising therapeutic paradigm against TNBC.

Given the lack of effective targeted therapies and the frequent chemoresistance observed in TNBC, [28] the dual induction of ferroptosis and mitophagy provides a novel therapeutic paradigm that may complement existing chemotherapy and immunotherapy regimens. Moreover, CISD1 expression could potentially serve as a predictive biomarker to identify patients most likely to benefit from xanthatin‐based treatment. Nevertheless, several limitations warrant consideration. First, while our in vitro and in vivo models consistently demonstrated anticancer efficacy, the present study did not evaluate the pharmacokinetics, tissue distribution, or long‐term toxicity of xanthatin in vivo; these parameters are essential for assessing druggability and will need to be systematically characterized in future studies. Second, although CISD1 was validated as the principal molecular target, it is plausible that xanthatin may interact with additional proteins, and such off‐target effects require further elucidation. Third, our findings are currently restricted to preclinical models, and validation in patient‐derived organoids or clinical samples will be crucial to confirm their clinical relevance. Looking ahead, structural optimization of xanthatin to improve bioavailability, combined with rational drug combinations targeting ferroptotic or mitochondrial pathways, may accelerate its translation into clinical practice and provide new therapeutic options for TNBC patients.

To summarize, our study demonstrates that the natural compound xanthatin exerts potent anticancer activity against TNBC by directly targeting and destabilizing CISD1. This posttranslational regulation of CISD1 disrupts iron homeostasis, thereby inducing ferroptosis, while simultaneously activating the PINK1/Parkin pathway to trigger mitophagy. These dual processes converge to amplify mitochondrial stress and suppress tumor progression. Importantly, xanthatin exhibited strong antitumor efficacy in vivo with minimal systemic toxicity, underscoring its translational potential. Collectively, our findings identify CISD1 as a novel mitochondrial vulnerability in TNBC and highlight xanthatin as a promising therapeutic candidate.

## 4 Materials and Methods

### 4.1 Reagents and Consumables

The following compounds were obtained from commercial sources: 500‐compound natural product library, xanthatin, acetylcysteine and MG‐132 from TargetMol (USA); Ferrostatin‐1, CCCP and deferoxamine B from Ambeed Corporation (Huston, TX, USA); Paclitaxel, doxorubicin, and cycloheximide from Selleck Chemicals (Huston, TX, USA); Chloroquine and 3‐methyladenine from MedChemExpress (USA). Agarose from Genefist (Shanghai, China). DNA gel extraction kits from Leagene Biotechnology (Beijing, China). Cell culture flasks and dishes from Guangzhou Jet Bio‐Filtration Co., Ltd (Guangzhou, China). Microcentrifuge tubes and conical centrifuge tubes from Promethe (Shanghai, China).

### 4.2 Cell Culture

MDA‐MB‐231 and MDA‐MB‐468 cell lines were maintained in Dulbecco's Modified Eagle Medium (DMEM, Gibco, USA) supplemented with 10% fetal bovine serum (FBS, ExCelll bio, Shanghai). BT‐549 and HEK293T cells were cultured in RPMI‐1640 medium containing 10% FBS. MCF10A cells were grown in commercially available specialized medium (CM‐0525, Procell Co., Ltd, Wuhan, China). Cell preservation was conducted using a serum‐free cryopreservation solution. (C40100, NCM Biotech, Suzhou, China). All cell lines were incubated at 37°C in a humidified atmosphere of 5% CO_2_.

### 4.3 Cell Viability Assay

Cells were seeded in 96‐well plates (Guangzhou Jet Bio‐Filtration Co., Ltd) and allowed to adhere overnight. Following treatment with designated concentrations of xantahtin or DMSO for specified durations, 10 µL of CCK‐8 reagent (APExBIO, Houston, USA) was added to each well. The plates were subsequently incubated at 37°C for 2 h. Absorbance was measured at 450 nm using a microplate reader (Tecan Infinite M200).

### 4.4 Cell Live/Dead Stain

The cell live/dead ratio was assessed using the calcein‐AM/propidium iodide (AM/PI) kit (KGA9501, KeyGEN BioTHCE). In brief, cells were seeded in 24‐well culture plates (Guangzhou Jet Bio‐Filtration Co., Ltd) and permitted 24 h adherence prior to xanthatin exposure. Following treatment, a freshly prepared calcein‐AM/PI work solution was introduced to each well. Incubation proceeded under physiological conditions (37°C, 5% CO_2_) for 30 min. After incubation, the cells were washed three times with Phosphate buffer saline (PBS, Servicebio, China) and then visualized using a fluorescence microscope (Guangzhou Yuntu Scientific Instrument Technology Co., Ltd).

### 4.5 EdU Proliferation Assay

Proliferative activity was evaluated using YSFluor 488 EdU Fluorescence Imaging & Flow Cytometry Assay kit (40275ES60, YEASEN Biochemical). Cells were plated in 24‐well plates and maintained under standard culture conditions. Following exposure to varying xanthatin concentrations, EdU working solution was introduced for 3 h. Specimens underwent fixation and permeabilization according to manufacturer specifications. Nuclei were counterstained with hoechst 33342 for 10 min. EdU‐positive cell percentages were quantified by fluorescence microscope (Guangzhou Yuntu Scientific Instrument Technology Co., Ltd).

### 4.6 Colony Formation Assay

Cells were seeded in 6‐well plates (Guangzhou Jet Bio‐Filtration Co., Ltd) at a density of 1000 cells per well. After adherence, cells were treated with xantahtin or DMSO, with the culture medium refreshed every 2–3 days. Following 10 days of incubation, cells were fixed with 4% paraformaldehyde (Leagene Biotechnology, Beijing, China) for 15 min and stained with 1% crystal violet (G1062, Solarbio Life Sciences Beijing, China) for 15 min at room temperature. Plates were rinsed with distilled water and air‐dried. Colonies were quantified manually.

### 4.7 RNA‐seq and KEGG Enrichment Analysis

MDA‐MB‐231 cells treated with 5 µm xanthatin or DMSO for 48 h were harvested for total RNA extraction. Subsequently, total RNA was sent to Wuhan Benagen Technology Co., Ltd for transcriptome sequencing. DEGs were identified with DESeq2 (v1.34.0) using thresholds of |log_2_FoldChange| ≥ 1 and adjusted *p*‐value < 0.05. The ClusterProfiler package of R (version 4.2.2) was used to perform KEGG pathway analysis.

### 4.8 Western Blot

Protein lysates were prepared using RIPA buffer (G2002, Servicebio, China) supplemented with phenylmethanesulfonyl fluoride (PMSF, G2008, Servicebio, China) and phosphatase inhibitors (NCM Biotech, Suzhou, China). Lysates were centrifuged at 12,000 × g for 15 min at 4°C, and supernatants were quantified via BCA assay (Thermo Fisher Scientific). Equal protein aliquots (20–30 µg) and pre‐stained protein marker (P9006, NCM Biotech, Suzhou, China) underwent separation on SDS‐polyacrylamide gels (PN3010, NCM Biotech, Suzhou, China) and electrophoretic transfer to PVDF membranes (Millipore). Membranes were blocked with 5% skim milk in TBST for 1 h at room temperature, followed by overnight incubation at 4°C with primary antibodies. After TBST washes, membranes were incubated with HRP‐conjugated secondary antibodies for 1 h. Protein bands were detected using Clarity Western ECL substrate (Abbkine Scientific Co., Ltd, China). β‐Actin served as the loading control for normalization. The antibodies used in the experiment include: β‐ACTIN (GB15003, 1:2000, Servicebio), SLC7A11 (T57046, 1:1000, Abmart), FTH1 (T55648, 1:1000, Abmart), TfR1 (T56618, 1:1000, Abmart), GPX4 (F1580, 1:1000, Selleck), CISD1 (#83775, 1:1000, CST), p62 (18420‐1‐AP, 1:4000, PTG), LC3 (A19665, 1:1000, Abconal), PINK1 (ER1706‐27, 1:1000, Huabio), Parkin (HA722952, 1:1000, Huabio), TOM20 (F0513, 1:1000, Selleck), Flag (F1804, 1:4000, Sigma).

### 4.9 JC‐1 Mitochondrial Membrane Potential Detection

Cells were seeded in 24‐well plates and treated with xanthatin or DMSO vehicle control for 48 h. Following treatment, mitochondrial membrane potential was evaluated using JC‐1 staining (KGA1904, KeyGEN BioTHCE) according to manufacturer guidelines. Briefly, cells were washed once with PBS before incubation with 300 µL JC‐1 working solution per well to ensure complete coverage. Specimens were maintained at 37°C in 5% CO_2_ for 30 min, then washed twice with pre‐warmed JC‐1 incubation buffer and immediately photographed under the fluorescence microscope.

### 4.10 ROS Detection

Intracellular ROS levels were quantified using the oxidation‐sensitive fluorescent probe DCFH‐DA (HY‐D0940, MedChemExpress). Cells cultured in 6‐well plates were treated with xanthatin or DMSO for 48 h. Following treatment, cells underwent triple washing with serum‐free DMEM and were incubated with 10 µm DCFH‐DA solution. Incubation proceeded at 37°C under 5% CO_2_ for 30 min. Cells were subsequently harvested, washed three times with PBS, and analyzed immediately using flow cytometry (BD Biosciences, San Jose, CA, USA).

### 4.11 Fe^2+^ Detection

Intracellular ferrous ion (Fe^2^^+^) levels were quantitatively assessed using the Fe^2^^+^‐specific fluorescent probe FerroOrange (HY‐K0322, MedChemExpress). Cells were seeded in 6‐well plates and treated with xanthatin for 48 h. Post‐treatment, cell pellets were collected and incubated with 300 µL serum‐free medium containing 1 µm FerroOrange working solution at 37°C under 5% CO_2_ for 30 min protected from light. Fluorescence intensity was then analyzed using a flow cytometer PE channel.

## 5 4‐HNE Detection

Cells were seeded in 6‐well plates and treated with xanthatin or DMSO for 48 h. Post‐treatment, cells were collected and washed with PBS followed by centrifugation (1000 rpm, 4°C, 5 min). Then, cell pellets were resuspended in 50 µL 4‐HNE antibody (A26085, 1:400, ABclonal) and incubated at room temperature for 30 min. Subsequently, the 4‐HNE antibody was washed away and a CoraLite488‐conjugated Goat Anti‐Rabbit IgG secondary antibody (SA00013‐2, 1:200, PTG) was added for a light‐protected incubation for 30 min. Finally, fluorescence signal analysis was conducted on the flow cytometer.

### 5.1 GSH and MDA Content Detection

GSH and MDA concentrations were quantified spectrophotometrically using GSH detection kits (BC1175, Solarbio Life Sciences Beijing, China) and MDA detection kits (BC0025, Solarbio Life Sciences Beijing, China) according to manufacturer specifications.

### 5.2 TEM

Cells treated with xanthatin or DMSO were fixed in 2.5% glutaraldehyde (4°C, 2 h). Samples underwent dehydration through an ethanol gradient (50% to 100%), infiltrated with acetone, and embedded in spurr embedding kit. Ultrathin sections (70 nm) were cut and mounted on copper grids. Images were acquired using a Hitachi HT7800 TEM.

### 5.3 Darts‐Ms

Cell lysates were prepared and partitioned into experimental (50 µm xanthatin) and control (equal volume of DMSO) aliquots. Following 1 h incubation at 25°C, samples underwent limited proteolysis with proteinase K (ST533, Beyotime, China) for 5 min at room temperature. Reactions were terminated with PMSF. The MS analysis was conducted at Shanghai Applied Protein Technology (Shanghai, China).

### 5.4 CETSA

Cell pellets were resuspended in PBS containing PMSF and lysed through three freeze‐thaw cycles in liquid nitrogen. The lysate was partitioned into two aliquots: one incubated with 50 µm xanthatin and the other with an equal volume of DMSO for 1 h at 25°C. Each aliquot was subdivided and exposed to incremental thermal denaturation (42, 46, 49, 52, 55, 58, 61,64, 67, and 70°C) for 3 min per temperature. Heat‐denatured aggregates were pelleted by centrifugation (12 000 × g, 10 min, 4°C). Soluble fractions were resolved by SDS‐PAGE, and target protein stability was analyzed via Western blot using CISD1 (#83775, 1:1000, CST) or Flag (F1804, 1:4000, Sigma) antibodies.

### 5.5 SPR

Binding kinetics between xanthatin and recombinant CISD1 protein (MedChemExpress) were assessed using a PlexArray HT A100 system. In brief, 3‐D photocrosslinked chips were activated with EDC/NHS, followed by covalent immobilization of 1 ug CISD1 protein. Afterward, different concentrations of the drugs were introduced to the chip surface at a predetermined flow rate to evaluate their binding affinity.

### 5.6 Molecular Docking

The protein structures were obtained from the AlphaFold Protein Structure Database (https://alphafold.ebi.ac.uk/). Molecular docking simulations were performed using AutoDock Tools and AutoDock Vina. The docking parameters were set as follows: a seed value of 10 000, an energy range of 3, and an exhaustiveness of 4. For each ligand‐protein pair, ten independent docking runs were conducted. The results were evaluated based on the calculated binding affinity (kcal/mol). The conformation with the lowest binding free energy was selected as the optimal binding pose for subsequent analysis. Visualization of the docked complexes was performed using PyMOL (v.3.1.3), and protein‐ligand interaction diagrams were generated using LigPlot^+^ (v.2.2).

### 5.7 MD Simulations

Because CISD1 is an outer mitochondrial membrane protein, the protein–ligand complex was embedded in a phospholipid bilayer prior to simulation. The orientation of the complex within the lipid bilayer was predicted using the PPM 2.0 web server (https://opm.phar.umich.edu/ppm_server2/), and the membrane system was subsequently constructed via CHARMM‐GUI (https://charmm‐gui.org/). The bilayer dimensions were set to 80 × 80 Å in the XY plane and composed of POPC and POPE lipids, with Na^+^ and Cl^−^ ions added to neutralize the system at a physiological salt concentration of 0.15 m. The force fields applied were AMBER19SB for proteins, Lipid17 for lipids, GAFF2 for ligands, and TIP3P for water. The system temperature was set at 310 K. All simulations were performed using GROMACS 2023.2. After energy minimization by the steepest descent algorithm for 5,000 steps, the system was equilibrated under the NVT ensemble by heating from 0 to 310 K, followed by equilibration under the NPT ensemble. Subsequently, a 100 ns production simulation was carried out at 310 K. Trajectories were analyzed for RMSD, RMSF, and hydrogen bond dynamics. Binding free energies were estimated using the Molecular Mechanics/Poisson–Boltzmann Surface Area (MM/PBSA) method, and per‐residue energy decomposition was performed to identify critical residues contributing to ligand binding.

### 5.8 Immunofluorescence Staining

Cells grown on cell culture coverslips (BS‐14‐RC, Biosharp, Hefei, China) were fixed with 4% paraformaldehyde for 15 min, permeabilized with immunostaining permeabilization solution (P0096, Beyotime, China) for 10 min, and blocked in 5% goat serum for 30 min. Primary antibodies diluted in blocking buffer were applied overnight at 4°C. After PBS washes, samples were incubated with CoraLite488‐conjugated Goat Anti‐Rabbit IgG secondary antibody (SA00013‐2, 1:200, PTG) for 1 h at 37°C protected from light. Nuclei were labeled with DAPI (P0131, Beyotime, China) for 5 min. Images were acquired using a LSM 800 confocal microscope (Carl Zeiss) with 63 × oil objective and analyzed via ZEN 3.7 (Blue edition).

### 5.9 Immunohistochemical Assay

A tissue microarray consisting of 48 paired TNBC tumor tissues and adjacent normal tissues was purchased from Shanghai Wellbio Biotechnology Co., Ltd. (Shanghai, China). Paraffin‐embedded tissue sections were first deparaffinized in xylene and rehydrated through a graded ethanol series. Antigen retrieval was performed by heating the slides in 0.1 m sodium citrate buffer (pH 6.0). Endogenous peroxidase activity was quenched by treating the sections with 3% hydrogen peroxide for 30 min, followed by blocking with 5% normal goat serum for 1 h at room temperature. The sections were then incubated overnight at 4°C with the following primary antibodies: Ki‐67 (TW0001, 1:250, Abmart), CISD1 (16006‐1‐AP, 1:1000, PTG), GPX4 (67763‐1‐Ig, 1:2000, PTG), 4‐HNE (A26085, 1:200, ABclonal), TfR1 (T56618, 1:200, Abmart) and LC3 (14600‐1‐AP, 1:300, PTG) antibodies. On the following day, a peroxidase‐conjugated avidin‐biotin complex and 3, 3‐diaminobenzidine (DAKO) were utilized as the chromogen, and the slices were then counterstained with hematoxylin. The stain intensity in the tissue microarray was assessed using a semi‐quantitative scoring system: 0 for no staining, 1 for moderate staining, 2 for weak staining, and 3 for strong staining. Based on this scale, specimens with scores of 0 or 1 were categorized as low expression, while those with scores of 2 or 3 were designated as high expression.

### 5.10 qPCR

Total RNA was extracted using the Total RNA Isolation Reagent Kit (Magen, China) according to manufacturer instructions. RNA concentration and purity were determined by spectrophotometry (NanoDrop, Thermo Scientific). cDNA synthesis was performed with 1 µg RNA using the TransGen Biotech Reverse Transcription Kit (Beijing, China). qPCR reactions contained 7.5 µL 2 × SYBR Green PCR Mix (Vazyme Biotech Co., Ltd), 0.5 µM gene‐specific primers, and 2 µL cDNA template in a 15 µL volume. Amplification was conducted on a QuantStudio 1 System (Applied Biosystems). Relative gene expression was normalized to GAPDH and calculated via the 2^−ΔΔCt^ method. Primer sequences are listed below: CISD1 (Forward primer: 5ʹ—GATCGCAGCAGTTACCATTGC – 3ʹ, Reverse primer: 5ʹ—GCATGTACTATCTTGGGGTTGTC – 3ʹ), Parkin (Forward primer: 5ʹ—GTGCAGAGACCGTGGAGAAA – 3ʹ, Reverse primer: 5ʹ—GCTGCACTGTACCCTGAGTT – 3ʹ), GAPDH (Forward primer: 5ʹ—CCATCACCATCTTCCAGGAG – 3ʹ, Reverse primer: 5ʹ—ATGATGACCCTTTTGGCTCC – 3ʹ).

### 5.11 Plasmid and Transfection

Lentiviral shRNA plasmids targeting CISD1 and Parkin (shCISD1#1: 5′‐ GATAAACCTTCACATCCAGAA ‐3′; shCISD1#2: 5′‐ GCTGCAATTGGTTATCTAGCT ‐3′; shParkin#1: 5′‐ TTGCACCTGATCGCAACAAAT ‐3′; shParkin#2: 5′‐ CTTAGACTGTTTCCACTTATA ‐3′) and non‐targeting control (shNC) were procured from Ruibiotech (Beijing, China). For overexpression, full‐length CISD1 cDNA was cloned into pcDNA3.1(+) with C‐terminal Flag tag. Lentivirus was produced in HEK293T cells by co‐transfecting transfer vectors with psPAX2 and pMD2.G packaging plasmids using NanoTrans Transfection Reagent 3000 (CT0006, CYTOCH, China). Viral supernatants were collected at 48 h post‐transfection. Target cells were transduced with virus in the presence of 10 µg/mL polybrene (BL628A, Biosharp, Hefei, China). Stable cell lines were selected with puromycin (BL528A, Biosharp, Hefei, China).

### 5.12 Coimmunoprecipitation Assay

Cells were lysed in IP buffer (P0013, Beyotime, China) supplemented with PMSF. Lysates were centrifuged at 12 000 × g for 15 min at 4°C. Supernatants were incubated overnight at 4°C with CISD1 antibodies (16006‐1‐AP, PTG). Protein A/G magnetic beads (HY‐K0202, MedChemExpress) were added to lysate‐antibody complexes and rotated for 4 h at 4°C. Beads were washed four times with cold PBST, resuspended in 2 × Laemmli buffer (G2075, Servicebio, China), and boiled at 95°C for 10 min. Immunoprecipitated proteins were analyzed by Western blot following standard protocols. Control samples used normal IgG (Santa Cruz, sc‐2025) to confirm binding specificity.

### 5.13 Animal Model

Female BALB/c mice (6 weeks old) were maintained under pathogen‐free conditions with 12‐h light/dark cycles. All procedures were approved by the Southern Medical University Animal Ethics Committee (SMUL202506061). For orthotopic TNBC models, 4T1 cells (1 × 10^7^) were implanted into the left mammary fat pads. After 7 days of palpable tumor formation, mice were randomized into three groups (n = 6/group): Vehicle control (0.5% CMC‐Na), Low‐dose xanthatin (10 mg/kg) and High‐dose xanthatin (20 mg/kg). Treatments were administered intraperitoneally every other day for 14 days. Tumor dimensions were measured every two days using calipers, with volume calculated as: V = (length × width^2^) / 2. Mice were sacrificed two days after the final dose. Tumors, major organs (liver, kidney, lung and spleen) and serum were harvested for subsequent analysis.

### 5.14 Statistical Analysis

All quantitative data are expressed as mean ± standard deviation (SD) from at least three independent biological replicates. Statistical comparisons between two groups used unpaired Student's T‐tests. Multiple group comparisons employed one‐way ANOVA with Tukey's post‐hoc test. Analyses were performed in GraphPad Prism 8.0 (GraphPad Software). Significance thresholds were defined as follows: ^*^for 0.01< *p* < 0.05, ^**^for 0.001< *p* < 0.01, and ^***^for *p* < 0.001.

## Author Contributions

Q.L. acquisition, analysis and interpretation of the data, statistical analysis, drafting the manuscript. H.C. and X.L. acquisition of the data, analysis and interpretation of the data. J.L., Y.L., Z.S., and S.G. technical and materials support. D.G., A.L., Q.D. funding acquisition, study concept and design, study supervision. All authors edited and approved the final manuscript.

## Funding

This work was supported by the National Natural Science Foundation of China (Grant Nos. 82503400, 32070676, 32370683), the China Postdoctoral Science Foundation (Grant No. 2025M78355), the National Postdoctoral Researchers Program (Grant No. GZC20251414), the Natural Science Foundation of Guangdong Province (Grant Nos. 2021A1515010737, 2023A1515012902), Theme‐based Research Scheme of Research Grants Council of Hong Kong SAR (T12‐201/20‐R, A.P.L.), the Guangdong Provincial Science and Technology Innovation Strategy Special Fund (Grant No. 2020B1212030006, A.P.L.), and the International Science and Technology Corporation Key Program of Jiangxi Province (Grant No. 20232BBH80012, A.P.L.).

## Conflicts of Interest

The authors declare no conflicts of interest.

## Ethics Approval and Consent to Participate

The Experimental Protocol For Animal Studies Was Reviewed and Approved By the Institutional Animal Care and Use Committee of Southern Medical University (SMUL202506061).

## Disclosure

The authors declare no conflicts of interest regarding the publication of this study.

## Consent for publication

Written informed consent for publication was obtained from all participants.

## Supporting information




**Supporting File 1**: advs74250‐sup‐0001‐SuppMat.pdf.

**Supporting File 2**: advs74250‐sup‐0002‐Supplementary Table S1.xlsx.

**Supporting File 3**: advs74250‐sup‐0003‐Supplementary Table S2.xlsx.

**Supporting File 4**: advs74250‐sup‐0004‐Supplementary Table S3.xlsx.

## Data Availability

The data that support the findings of this study are available from the corresponding author upon reasonable request.
