# Supplementary material for: Xanthatin Targets CISD1 to Drive Ferroptosis and Mitophagy as a Dual Anticancer Strategy in Triple‐Negative Breast Cancer
Source: Adv Sci (Weinh). 2026 Feb 6;13(21):e20051. doi: 10.1002/advs.202520051 (PMC13073315; doi:10.1002/advs.202520051)
Supplement: Supplementary file 1 — Supporting File 1: advs74250‐sup‐0001‐SuppMat.pdf. [file ADVS-13-e20051-s004.docx]

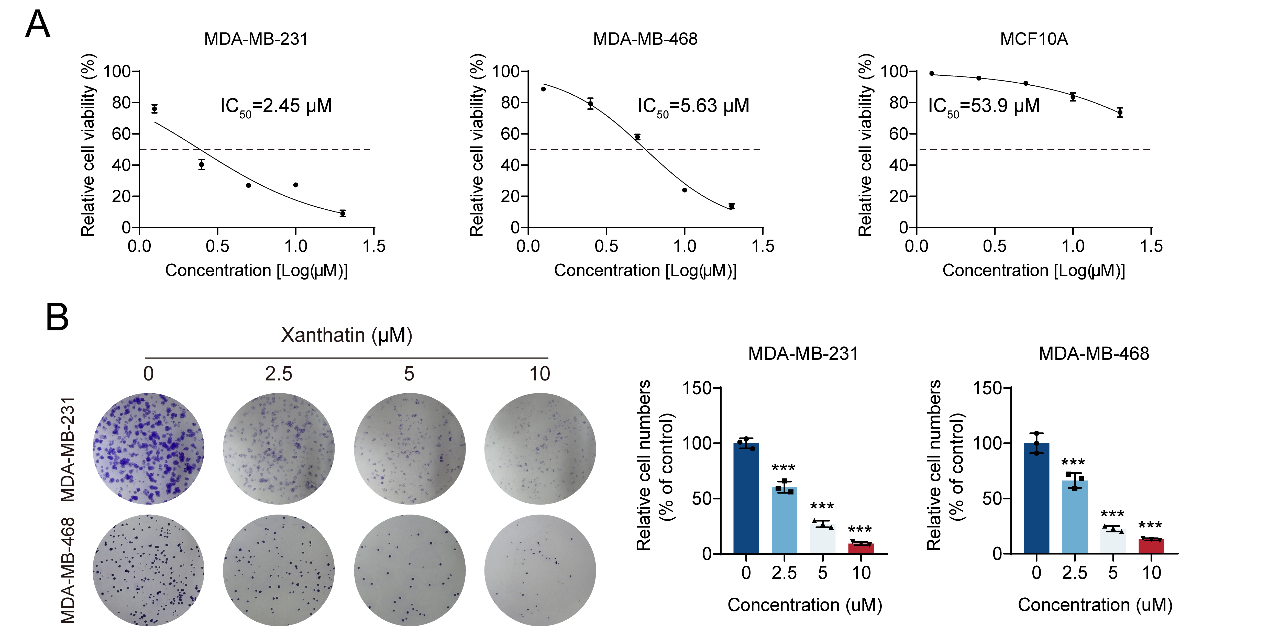


**Supplementary Figure S1.** (A) IC_50_ of xanthatin in MDA-MB-231, MDA-MB-468, and MCF10A cells. (B) Representative images and quantification of colony formation assays in MDA-MB-231 and MDA-MB-468 cells treated with xanthatin at the indicated concentrations for 10 days. Bars, SDs; * 0.01 < *P* < 0.05, ** 0.001 < *P* < 0.01, and *** *P* < 0.001.


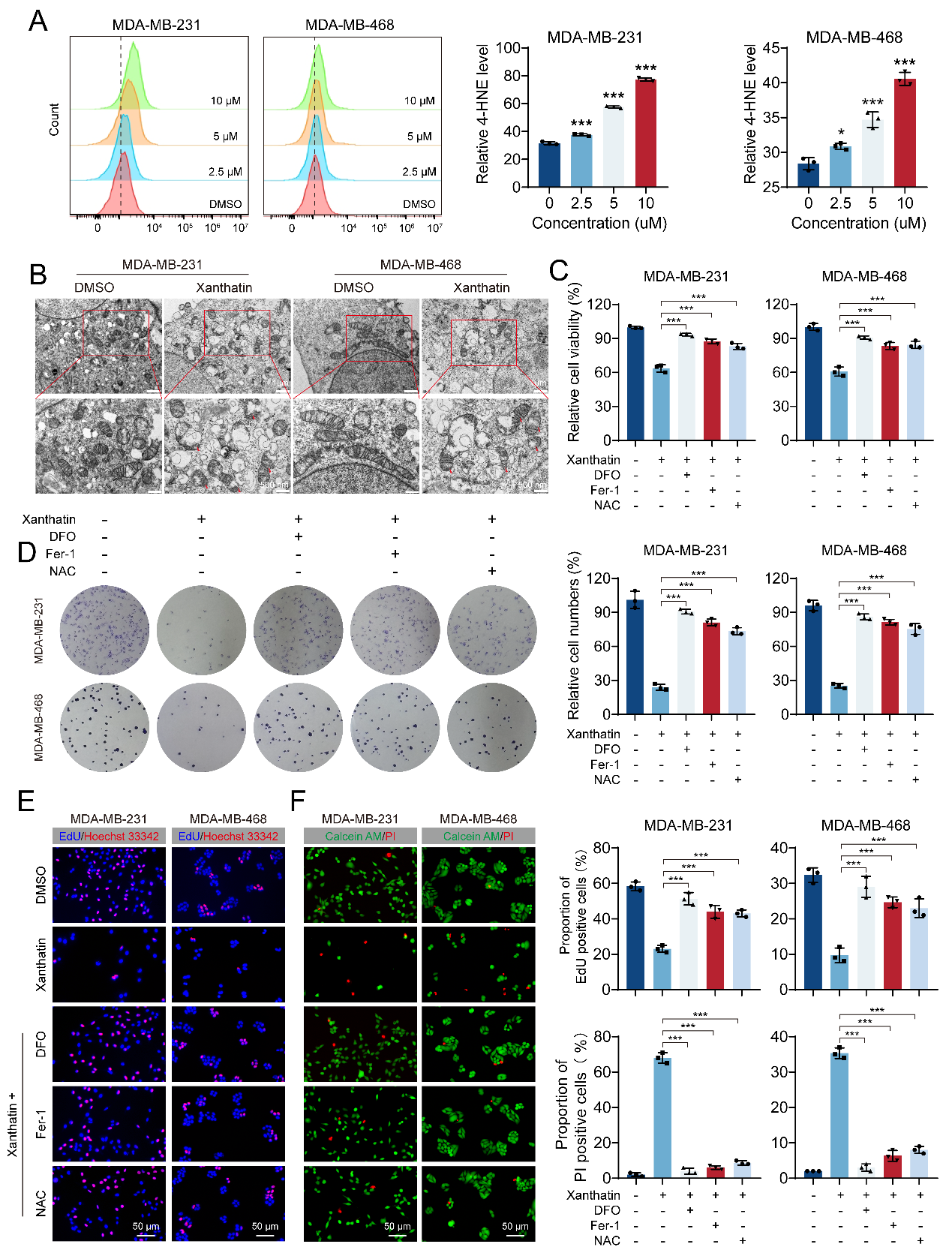


**Supplementary Figure S2.** (A) Flow cytometry was used to detect 4-HNE levels in MDA-MB-231 and MDA-MB-468 cells after treatment with xanthatin for 48 h. (B) TEM images of MDA-MB-231 and MDA-MB-468 cells treated with xanthatin for 48 h. Scale bar = 1 μm (upper panels), 500 nm (lower panels). (C–F) Cells were pretreated with 20 μM DFO or 1 μM Fer-1 or 5 mM NAC and then exposed to xanthatin for 48 h. Cell viability was determined by CCK-8 (C); proliferation capacity was evaluated by EdU incorporation (D); colony formation ability was assessed by colony formation assay (E); and cell survival was examined by calcein-AM/PI staining (F). Scale bar = 100 μm. Bars, SDs; * 0.01 < *P* < 0.05, ** 0.001 < *P* < 0.01, and *** *P* < 0.001.


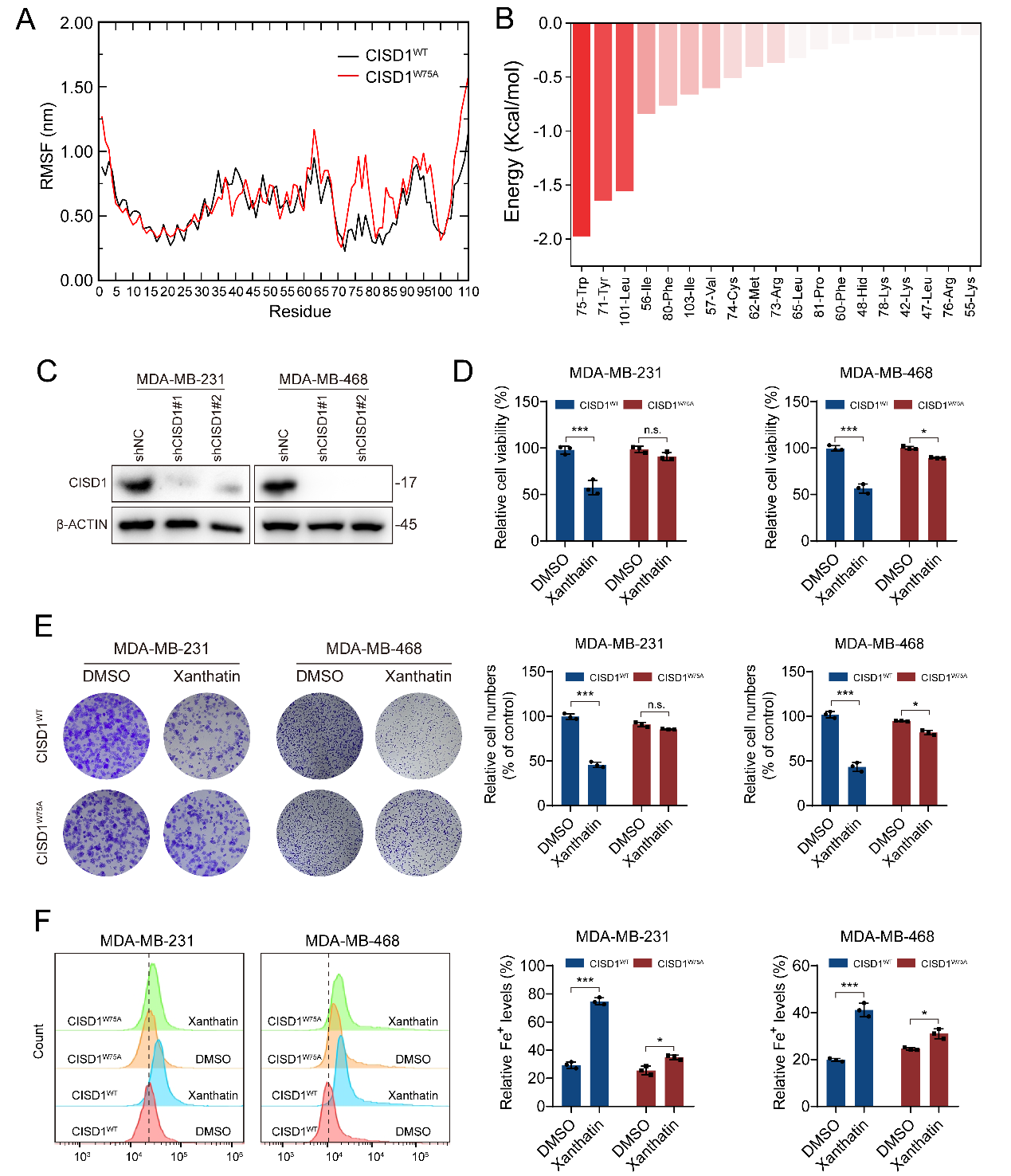


**Supplementary Figure S3.** (A) RMSF profiles of wild-type and mutant CISD1, indicating increased flexibility around the mutation site. (B) Per-residue energy contribution analysis highlighting Trp-75 as the dominant binding hot spot, together with Tyr-71, Leu-101, and Ile-56. (C) Western blot analysis of CISD1 expression in MDA-MB-231 and MDA-MB-468 stable knockdown cell lines (shCISD1#1, shCISD1#2) generated by lentiviral infection compared with negative control (shNC). (D) Cell viability and colony formation assays in shCISD1#1 cells reconstituted with CISD1^WT^ or CISD1^W75A^ plasmids, followed by treatment with DMSO or xanthatin. (F) Flow cytometric analysis and quantification of intracellular Fe²⁺ levels in shCISD1#1 cells reconstituted with CISD1^WT^ or CISD1^W75A^ plasmids and then treated with xanthatin for 48 h.


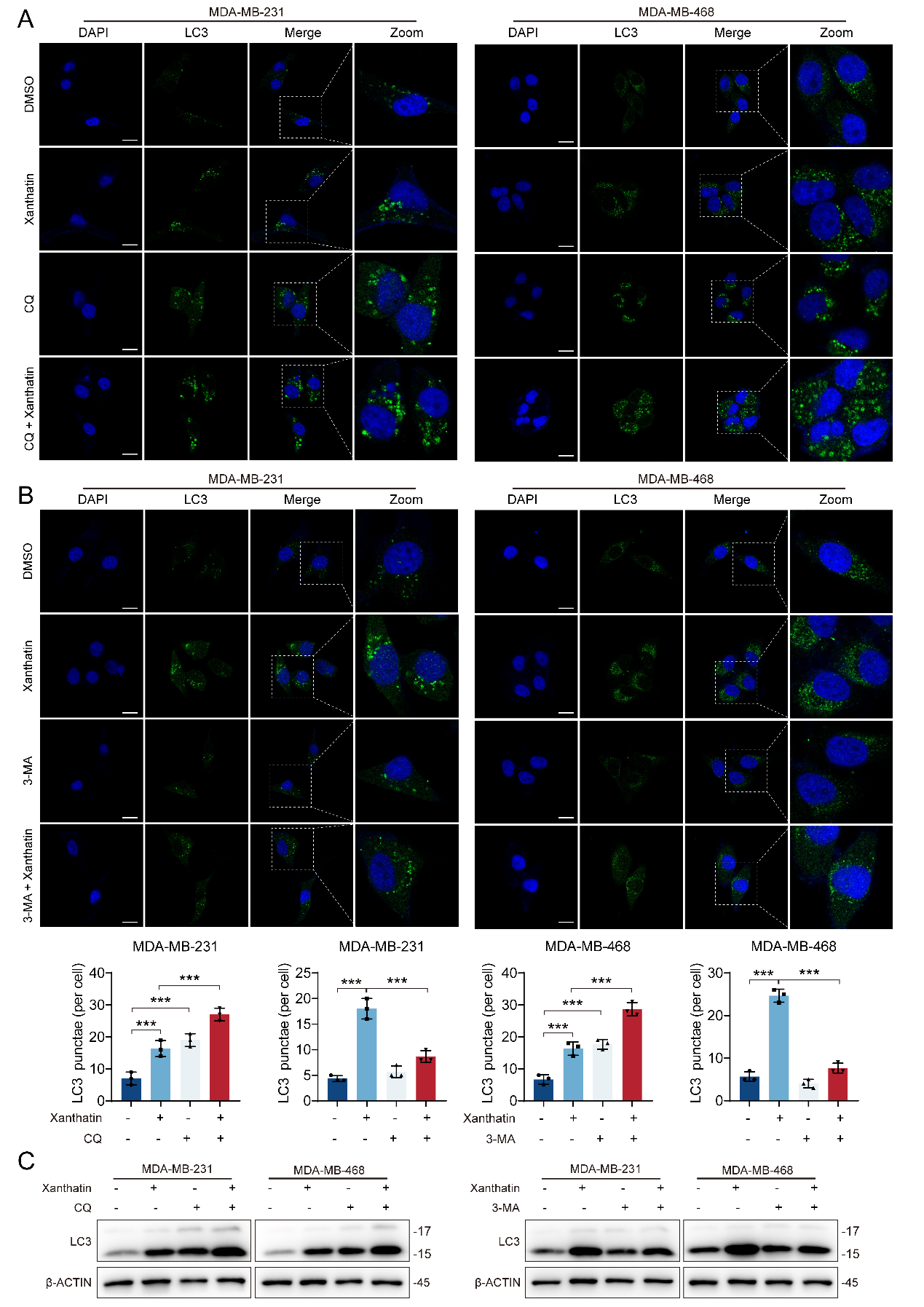


**Supplementary Figure S4.** (A) Immunofluorescence staining of LC3 in TNBC cells treated with xanthatin for 48 h, with or without CQ pretreatment (20 µM, 12 h). Scale bar = 20 μm. (B) Immunofluorescence staining of LC3 in TNBC cells treated with xanthatin for 48 h, with or without 3-MA pretreatment (1 mM, 12 h). Scale bar = 20 μm. (C) Western blot analysis of LC3 in TNBC cells treated with xanthatin for 48 h, with or without CQ (20 µM, 12 h) or 3-MA (1 mM, 12 h) pretreatment. Bars, SDs; * 0.01 < *P* < 0.05, ** 0.001 < *P* < 0.01, and *** *P* < 0.001.


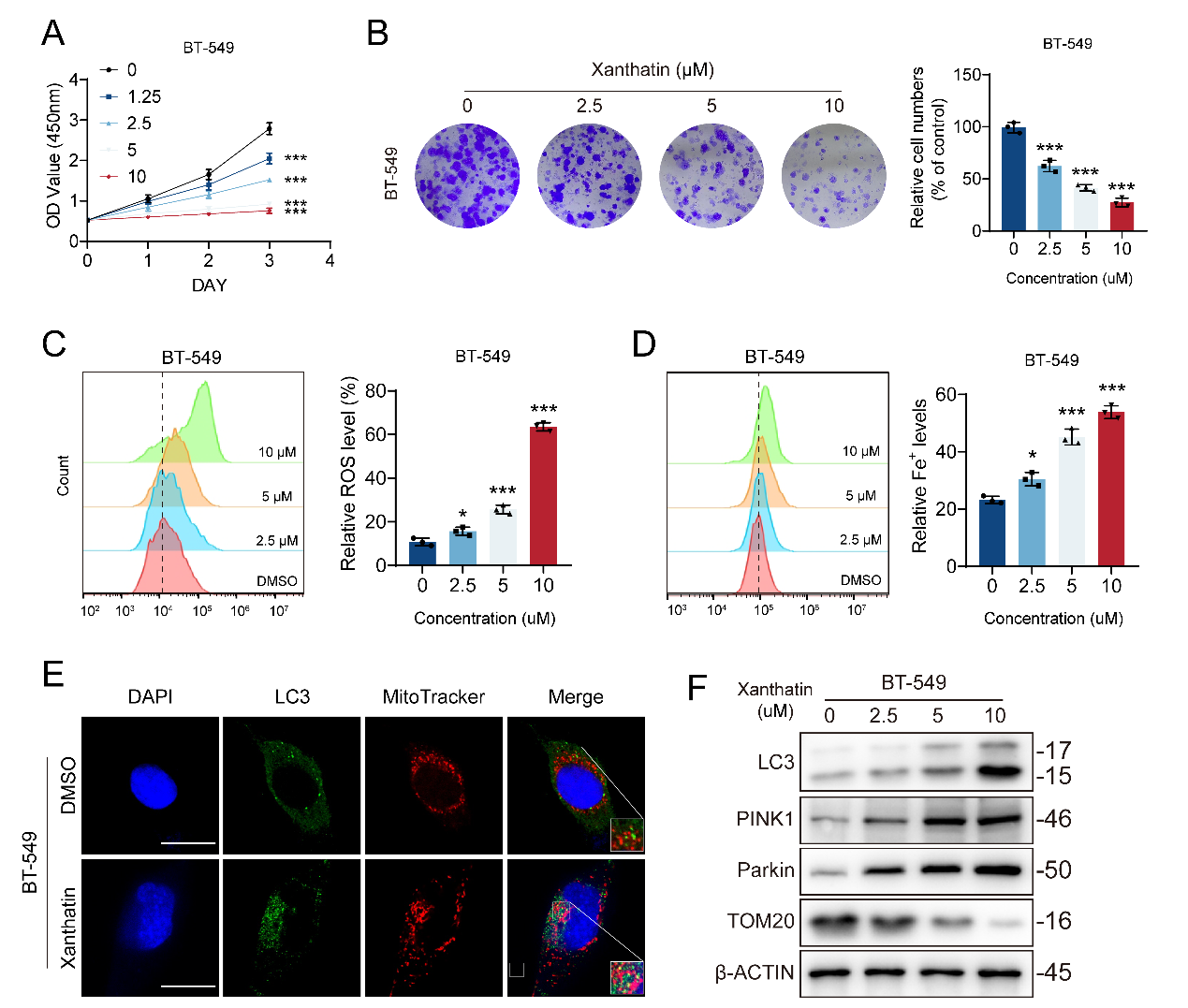


**Supplementary Figure S5.** (A) Growth curves of BT-549 cells treated with the indicated concentrations of xanthatin for indicated time. (B) Representative images and quantification of colony formation assays in BT-549 cells treated with xanthatin at the indicated concentrations for 10 days. (C-D) ROS and Fe²⁺ levels were determined by flow cytometry in BT-549 cells after xanthatin treatment for 48 h. (E) Immunofluorescence staining of LC3 and MitoTracker in BT-549 cells treated with xanthatin for 48 h. Scale bar = 20 μm. (F) Western blot analysis of autophagy- and mitophagy-related proteins LC3, PINK1, Parkin, and TOM20 in BT-549 cells after treatment with xanthatin for 48 h. Bars, SDs; * 0.01 < *P* < 0.05, ** 0.001 < *P* < 0.01, and *** *P* < 0.001.


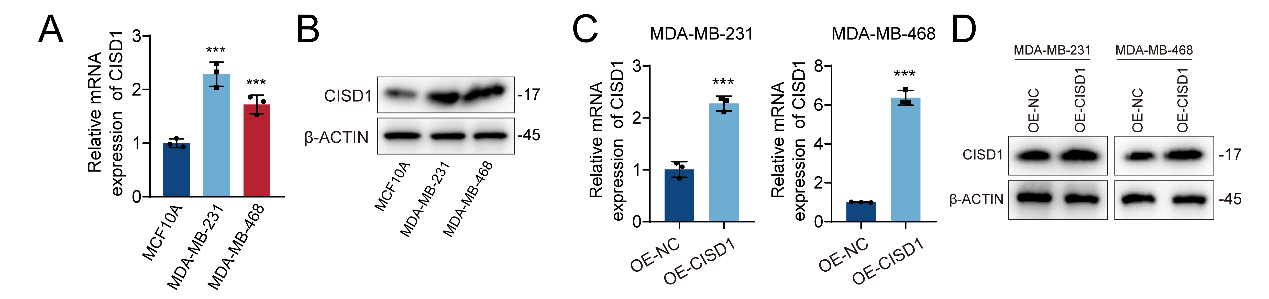


**Supplementary Figure S6.** (A-B) qPCR (A) and Western blot (B) analysis of CISD1 expression in mammary epithelial cells (MCF10A) and TNBC cells (MDA-MB-231, MDA-MB-468). (C-D) qPCR (C) and Western blot (D) analysis of CISD1 expression in MDA-MB-231 and MDA-MB-468 stable cell lines with CISD1 overexpression (OE-CISD1) generated by lentiviral infection compared with negative control (OE-NC). Bars, SDs; * 0.01 < *P* < 0.05, ** 0.001 < *P* < 0.01, and *** *P* < 0.001.


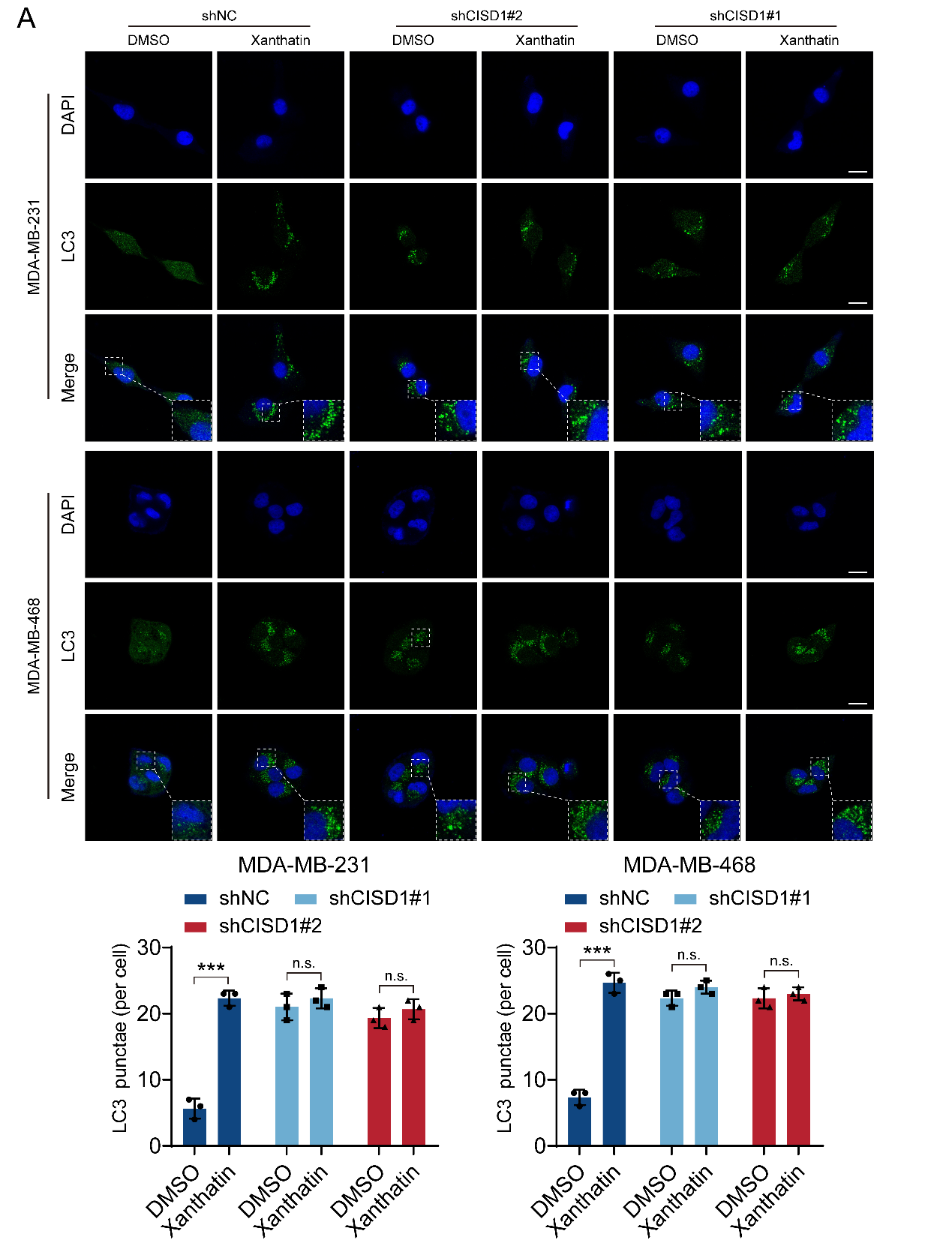


**Supplementary Figure S7.** (A) Immunofluorescence staining of LC3 in MDA-MB-231 and MDA-MB-468 stable knockdown cell lines (shCISD1#1, shCISD1#2) and control cells (shNC) treated with DMSO or xanthatin for 48 h. Representative images are shown, and quantification of LC3 puncta per cell is presented. Scale bar = 20 μm. Bars, SDs; * 0.01 < *P* < 0.05, ** 0.001 < *P* < 0.01, and *** *P* < 0.001. n.s., no significance.
